# Adaptive Control of Lower-Limb Assistive Exoskeleton for Rehabilitation Using Deep Reinforcement Learning

**DOI:** 10.3390/s26165217

**Published:** 2026-08-17

**Authors:** Ali Foroutannia, Masoud Mohammadian, Kumudu Munasinghe

**Affiliations:** Faculty of Science and Technology, University of Canberra, Canberra, ACT 2617, Australia; masoud.mohammadian@canberra.edu.au (M.M.); kumudu.munasinghe@canberra.edu.au (K.M.)

**Keywords:** biomechanical modelling, deep reinforcement learning, lower-limb rehabilitation exoskeleton, noise robustness, robust assistance adaptive control, stroke rehabilitation

## Abstract

Lower-limb rehabilitation exoskeletons have the potential to improve gait recovery after stroke by providing intensive and repetitive training. However, conventional control strategies often rely on fixed control parameters and exhibit limited adaptability to patient-specific characteristics, sensor noise, and dynamic uncertainties. This paper proposes an adaptive control framework that combines deep reinforcement learning (RL) with model-based impedance control for personalised lower-limb exoskeleton assistance. Patient-specific biological parameters are incorporated into the simulation environment and reward formulation to improve adaptability and robustness. Three state-of-the-art deep RL algorithms, Deep Deterministic Policy Gradient (DDPG), Twin Delayed Deep Deterministic Policy Gradient (TD3), and Soft Actor-Critic (SAC), are evaluated in a continuous control environment under varying signal-to-noise ratio (SNR) conditions ranging from 5 dB to noise-free conditions. Results demonstrate that TD3 achieves the most stable learning performance, obtaining a mean reward of −354.24 under noise-free conditions, while DDPG provides the highest joint-angle tracking accuracy with an RMSE of 0.0369 rad. SAC exhibits superior robustness in noisy environments, achieving the highest learning ratio of 0.51 at 5 dB SNR. Furthermore, the proposed personalised framework reduces tracking errors by up to 27% compared with non-personalised baseline approaches. The findings indicate that integrating patient-specific information with RL-based adaptive control can significantly enhance robustness, tracking performance, and personalisation in exoskeleton-assisted gait rehabilitation, providing a promising direction for future intelligent rehabilitation systems.

## 1. Introduction

Stroke is a principal cause of long-term impairment and diminished quality of life internationally, ranking as the second greatest cause of mortality and a significant contributor to disability-adjusted life years (DALYs) worldwide [1,2]. In 2021, around 11.9 million new strokes and over 93 million existing cases were documented globally, with forecasts suggesting further increases attributed to ageing demographics and escalating risk factors in various areas [2,3]. In Australia, the burden is significant: recent estimates indicate 41,100 to 45,785 stroke incidents annually (about 113 events per day, or one every 11 min), with a considerable number being first-ever strokes, and over 440,000 individuals enduring the aftermath of stroke. A considerable proportion of survivors exhibit pronounced lower-limb motor impairments, leading to abnormal gait patterns, diminished walking speed and endurance, balance deficiencies, heightened fall risk, reliance on assistance for everyday activities, and considerable emotional consequences [4,5].

Effective post-stroke rehabilitation emphasises the restoration of motor function using high-repetition, task-specific training to enhance neuroplasticity and facilitate gait recovery. However, conventional therapist-administered rehabilitation is resource-expensive, constrained by staffing shortages and therapist fatigue, and frequently fails to provide the intensive dosage necessary for significant functional improvements [6,7].

Robotic lower-limb assistive exoskeletons have evolved as a promising solution to address these challenges. These devices offer mechanical support to the hip, knee, and ankle joints, enabling consistent and repetitive practice of functional tasks such as walking, sit-to-stand transitions, and standing balance exercises while reducing the physical burden on therapists and allowing prolonged training sessions. Recent advances in wearable robotic systems have demonstrated the potential of lightweight and compliant assistive devices for gait rehabilitation and mobility enhancement. For example, Ma et al. developed a reconfigurable exomuscle system based on pneumatic artificial muscles capable of providing both hip-flexion and ankle-plantarflexion assistance while reducing metabolic cost during walking [8]. Similarly, Watanabe et al. proposed a sensor-minimal pneumatic exosuit that integrates posture estimation and gait assistance through phase-dependent role switching within a unified pneumatic architecture [9]. Further, devices such as the Lokomat, Ekso GT, and ReWalk have demonstrated efficacy in clinical settings for improving gait metrics and patient engagement [3,10,11,12]. These studies demonstrate the growing interest in adaptive and human-centred exoskeleton technologies. Despite these advances, most existing studies primarily focus on hardware design, actuation strategies, and gait-phase assistance. In contrast, the present study investigates adaptive high-level control through deep reinforcement learning combined with impedance control.

Despite these advancements, control strategies for exoskeletons remain a major challenge. Model-based approaches, such as PID controllers and impedance/admittance control, rely on precise mathematical models of the human–exoskeleton system but struggle with real-world uncertainties, including inter-subject variability (e.g., differences in bone length, muscle mass, joint stiffness, spasticity, or fatigue), unmodelled dynamics, and external disturbances [12,13,14]. Model-free machine learning methods often require large labelled datasets and generate generalised policies that fail to accommodate individual patient needs, resulting in suboptimal performance and limited compliance [15,16,17]. For example, supervised sequence-learning approaches such as Long Short-Term Memory (LSTM) networks and Time Delay Neural Networks (TDNNs) have shown promising performance in predicting gait trajectories and joint kinematics, and their primary objective is to approximate temporal patterns from historical observations [18]. Consequently, these methods typically operate as reactive predictors and may exhibit limited adaptability when system dynamics, environmental conditions, or patient characteristics differ from those encountered during training.

In contrast to conventional supervised sequence-learning approaches that primarily predict future trajectories from historical observations, reinforcement learning (RL) learns a control policy through continuous interaction with the environment and explicitly optimises long-term cumulative rewards. This enables the controller to account for the future consequences of current actions, adapt to disturbances and uncertainties, and develop decision-making strategies that generalise across varying rehabilitation conditions. The ability to learn adaptive and goal-directed behaviours makes RL particularly suitable for human–exoskeleton interaction, where patient-specific variability, changing biomechanical conditions, and dynamic uncertainties are unavoidable. Furthermore, deep reinforcement learning (DRL) extends these capabilities to high-dimensional continuous state-action spaces by combining reinforcement learning principles with deep neural networks. Through trial-and-error learning and reward maximisation, DRL algorithms can effectively address complex control problems involving non-linear dynamics, sensor noise, and environmental variability [19,20,21,22]. In particular, state-of-the-art continuous-control algorithms such as Deep Deterministic Policy Gradient (DDPG), Twin Delayed Deep Deterministic Policy Gradient (TD3), and Soft Actor-Critic (SAC) have demonstrated considerable success in robotic applications due to their ability to provide stable learning, robust decision-making, and improved adaptability under uncertain operating conditions [23,24].

This paper proposes a novel adaptive control system for lower-limb assistive exoskeletons that integrates model-based impedance control with deep reinforcement learning to deliver tailored and robust assistance for key rehabilitation tasks (walking, sitting, and standing). The primary innovation is the explicit incorporation of patient-specific biological parameters (bone length and muscle mass) into the RL state space and reward function, enabling adaptive responses to uncertainty, noise, and inter-subject variability. Three prominent reinforcement learning algorithms (DDPG, TD3, and SAC) are systematically evaluated in a simulated continuous action space under different signal-to-noise ratios. Results demonstrate that TD3 provides superior stability and resilience. While the current study employs a simplified dynamic model to systematically evaluate the RL algorithms, future extensions will address more realistic human–exoskeleton interactions.

The remainder of this paper is organised as follows: Section 2 reviews the related literature on exoskeleton control and reinforcement learning applications. Section 3 presents the proposed methodology, including the musculoskeletal model, reinforcement learning formulation, and simulation setup. Section 4 reports the experimental results and comparisons. Section 5 discusses the implications, limitations, and future directions.

## 2. Literature Review

Lower-limb exoskeletons have evolved significantly since their introduction in the 1960s, initially developed for military applications and later adapted for rehabilitation of gait disorders such as stroke [25]. Early systems primarily focused on mechanical design to enhance mobility, exemplified by the Berkeley Lower Extremity Exoskeleton (BLEEX), which emphasised passive assistance [26,27]. Over the past decade, research has shifted toward active control strategies aimed at improving human–robot interaction, reducing therapist workload, and enabling personalised treatment [28]. This study categorises existing control approaches into model-based and model-free techniques, with a particular focus on reinforcement learning (RL) for adaptive control, and identifies the limitations that the proposed framework seeks to overcome.

### 2.1. Model-Based Control

Conventional model-based controllers rely on explicit mathematical models of the human–exoskeleton system. PID controllers have been widely employed for trajectory tracking in devices such as the Lokomat, providing consistent support during gait training [29]. Impedance and admittance control improve compliance by modelling the system as a mass-spring-damper, allowing adjustable stiffness based on user intent [30]. Cao et al. incorporated musculoskeletal models into adaptive impedance control to account for biological factors such as muscle length, achieving reduced tracking errors in simulation [31]. However, these methods are sensitive to model inaccuracies, including unmodelled uncertainties (e.g., joint friction and patient variability), leading to degraded performance in real-world conditions. They also often require extensive parameter tuning for each patient, which limits scalability [32].

### 2.2. Model-Free Control

To overcome the limitations of model-based approaches, model-free machine learning techniques have gained increasing attention [33]. Supervised learning methods, such as neural networks for gait prediction, have been used to map sensor data to joint torques [15]. Unsupervised techniques, including clustering for gait phase detection, enable real-time adaptation without labelled data [34]. Nevertheless, supervised approaches typically demand large datasets, while unsupervised methods often lack goal-directed optimisation, resulting in suboptimal performance in highly dynamic environments [18,35].

### 2.3. Reinforcement Learning

Reinforcement learning has emerged as a powerful paradigm for exoskeleton control, offering individualised adaptation and robustness to uncertainty in human–exoskeleton interaction (HEI). This section reviews recent advances in RL-based exoskeleton control and highlights current limitations [36,37,38].

Early RL applications focused on specific tasks such as squatting and walking. Luo et al. developed an RL controller for stable squatting by incorporating centre of pressure (CoP) into the reward function and using dynamic randomisation and adversarial perturbations to improve robustness against unexpected interaction forces [39]. Huang et al. proposed a hierarchical interactive learning (HIL) framework combining dynamic movement primitives (DMPs) with RL, achieving a 12% reduction in human–exoskeleton interaction forces across different terrains [40]. Although promising, these methods were often limited to predefined movements and showed limited generalisation across patients [39,40].

Recent studies have leveraged deep RL algorithms to handle continuous action spaces and complex dynamics. Luo et al. introduced a decoupled RL framework with three neural networks (policy, interaction force prediction, and muscle coordination) trained via domain randomisation, enabling effective walking assistance for both quadriplegic and hemiplegic patients without manual parameter tuning [41]. Zheng et al. applied deep RL for sensitivity amplification and virtual impedance control, reducing human–exoskeleton interaction forces by up to 46% compared to conventional methods [36,42,43]. Other works have integrated DMPs with RL for joint trajectory learning [44] and variational autoencoders (VAE) with RL for adaptive gait prediction [45].

Advanced on-policy algorithms such as PPO have also been explored due to their stability and sample efficiency. Dizor et al. combined long short-term memory (LSTM) networks with PPO for movement intention prediction using surface electromyography (sEMG) and inertial measurement unit (IMU) data [46]. Mowbray and Rakshit utilised PPO with model-based virtual planning for improved trajectory tracking [47]. Wong Sang et al. investigated offline RL using TD3 with behaviour cloning to enhance gait tracking while addressing safety during training [37]. These studies highlight RL’s ability to manage high-dimensional spaces and reduce reliance on precise dynamic models [37,44].

### 2.4. Research Gaps and Motivation

Despite these achievements, several limitations remain. Most RL-based controllers rely on generalised training data, offering limited personalisation with respect to individual biological factors such as bone length or muscle mass [40,41]. Real-time adaptation to dynamic human–exoskeleton interaction forces and environmental changes (e.g., terrain transitions) remains computationally intensive [36,42,43]. Although algorithms such as TD3 and PPO show noise resilience [37,41], there is a lack of comprehensive comparative studies evaluating their performance in continuous action spaces under varying uncertainty levels. Moreover, many approaches are task-specific (e.g., squatting [39] or walking [41,44]) and struggle to generalise to other essential activities such as sitting and standing. Safety concerns in online RL training further hinder real-world deployment [37].

Recent related works include Xu et al. [48], who proposed a mirror adaptive impedance control using reinforcement learning for a multi-mode soft exoskeleton, and Xu et al. [49], who developed a DMP-based motion generation scheme for robotic mirror therapy with RL-optimised impedance parameters. Tu et al. [50] presented a data-driven RL framework for optimal and adaptive personalisation of a hip exoskeleton. While these studies advance RL application in rehabilitation robotics, our work differs by integrating patient-specific biological parameters directly into the state space and reward function of a hybrid impedance–RL controller for a bilateral lower-limb exoskeleton and by providing a systematic comparison of three deep RL algorithms (DDPG, TD3, and SAC) under controlled noise conditions and domain randomisation.

The proposed framework addresses these gaps by combining deep RL algorithms with model-based impedance control and incorporating patient-specific biological factors to achieve robust, personalised adaptive control for stroke rehabilitation. This study aims to identify the most effective RL method through comprehensive evaluation in a simulated continuous action space with varying noise levels.

## 3. Methodology

This section outlines the proposed adaptive control framework for the lower-limb exoskeleton, integrating a musculoskeletal model with RL algorithms. The methodology is designed to enable patient-specific adaptation, handling uncertainties such as noise and biological variations. Simulations were performed using MATLAB R2025a with the reinforcement learning toolbox and real human gait data from the standard dataset included in our recent paper. The framework consists of three main components: the musculoskeletal model, RL algorithms, and the simulation environment.

### 3.1. Overview of the Proposed Method

**Figure 1:**
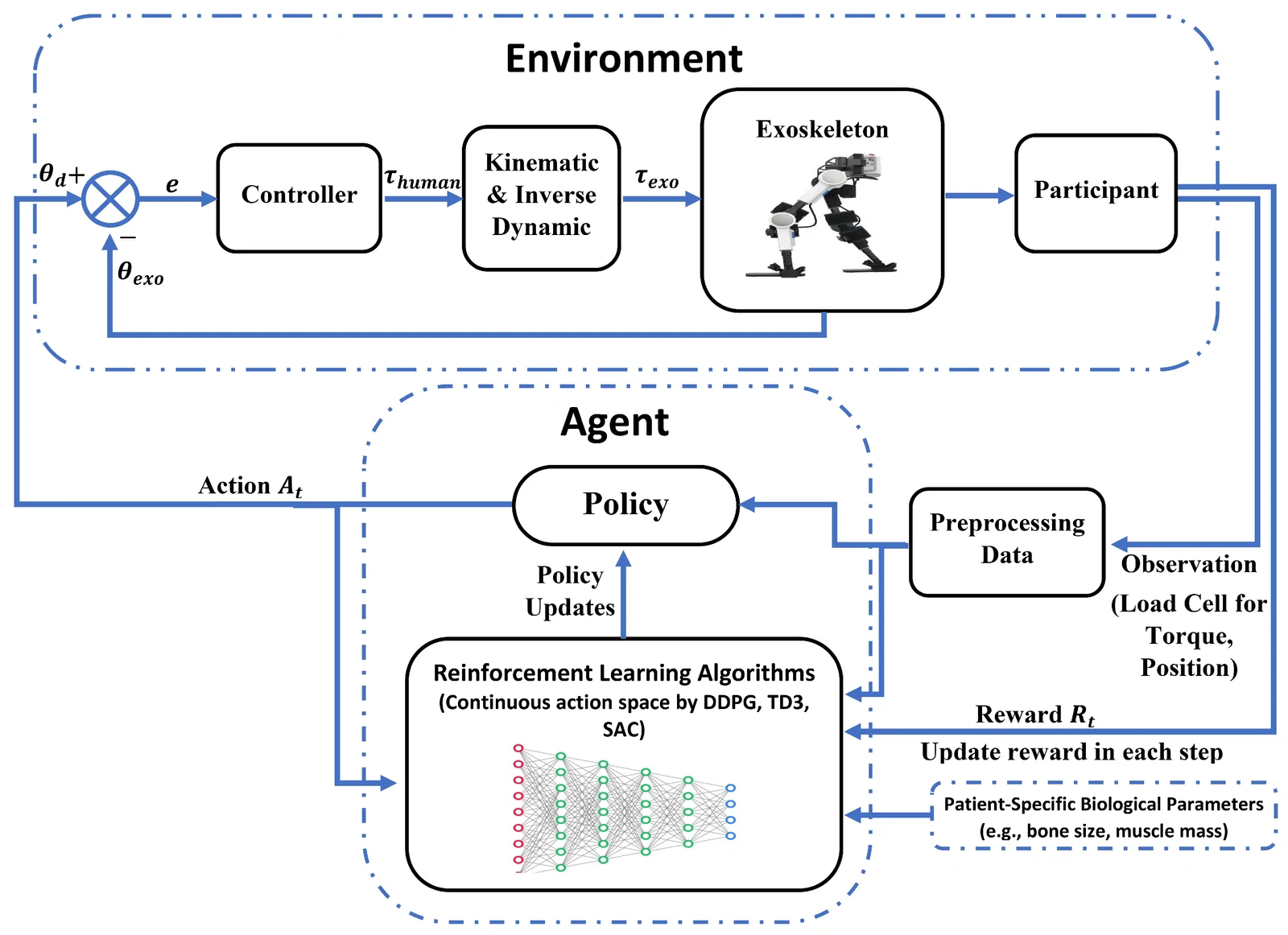
Schematic of the proposed adaptive control framework, integrating RL algorithms (DDPG, TD3, SAC) with model-based controllers to personalise lower-limb exoskeleton behaviour for walking, sitting, and standing, utilising sensory data and kinematics and inverse dynamics. Patient-specific biological parameters enter the control loop through the RL agent’s state space and reward function, enabling sequential decision-making tailored to individual variability, uncertainty, and long-term rehabilitation benefits.

The proposed system, illustrated in Figure 1, consists of two main components: the Environment and the Agent. The environment includes the exoskeleton, the human participant, and a controller that converts human intent ($\theta_{\mathrm{human}}$) into desired joint angles ($\theta_{\mathrm{exo}}$) using kinematics and inverse dynamics. The controller minimises the tracking error (*e*) between the desired and actual joint positions, generating appropriate commands for the actuators. Sensor data from load cells (torque and position) are preprocessed to form observations that capture real-time human–robot interaction dynamics. The Agent comprises a policy network and reinforcement learning algorithms (DDPG, TD3, and SAC) operating in a continuous action space. The policy generates actions ($A_{t}$) based on the preprocessed observations, while the RL algorithms iteratively update the policy to maximise the accumulated reward ($R_{t}$). The RL agent receives a four-dimensional state consisting of the bilateral hip and knee joint angles. Additional variables, including joint velocities, accelerations, and patient-specific biological parameters, are maintained within the simulation environment and are used for dynamic modelling, impedance control, reward computation, and parameter randomisation. The reward function is designed to penalise tracking errors, torque inconsistencies, and abrupt movements while encouraging stability and successful task completion. As shown in Figure 1, patient-specific biological parameters are explicitly incorporated into both the state space, simulation environment, and the reward function. This integration enables the RL agent to learn personalised control policies that adapt to individual variability and uncertainties, distinguishing the proposed framework from conventional uniform control strategies. Training is performed using recorded gait data augmented with domain randomisation to simulate biological variations and sensor noise. Through repeated episodes, the policy is refined based on the received rewards, allowing the agent to adapt to changing patient conditions and develop experiential memory for repetitive movements. Although the RL agent primarily learns to track reference trajectories derived from recorded human gait data, the inclusion of randomised biological parameters and noise in the state and reward function enables the policy to adapt to patient-specific variability and uncertainties, moving beyond pure trajectory tracking toward personalised assistance. The overall objective is to develop a resilient, patient-specific controller that overcomes the limitations of traditional methods, which lack personalisation and robustness to real-world uncertainties.

### 3.2. Dataset

A gait dataset collected from seven healthy subjects was used for training and evaluating the proposed reinforcement learning framework. The data, originally gathered in our previous work [18], include signals acquired from inertial measurement units (IMUs), force-sensitive resistors (FSRs), load cells, and joint position sensors sampled at 500 Hz. All participants had no history of neurological or psychiatric disorders and exhibited no motor impairments such as hemiplegia or hemiparesis. The data collected from the seven healthy participants were divided into training, validation, and testing subsets, comprising 70%, 15%, and 15% of the dataset, respectively. The experimental protocol consisted of treadmill walking trials performed at five different speeds (0.3, 0.4, 0.5, 0.6, and 0.85 m/s). To capture dynamic gait transitions, treadmill speed was gradually increased from 0.3 m/s to 0.85 m/s and subsequently decreased back to 0.3 m/s (0.3, 0.4, 0.5, 0.6, 0.85, 0.85, 0.6, 0.5, 0.4, and 0.3 m/s). Each trial lasted 80 s, comprising 10 s of standing before walking, 60 s of treadmill walking, and 10 s of standing after the trial. The inclusion of multiple walking speeds enabled the dataset to capture gait variability under different locomotion conditions and provided a basis for developing speed-independent control policies.

This study was conducted in accordance with the Declaration of Helsinki (2008), Good Clinical Practice (GCP) guidelines, and relevant ethical regulations. All participants provided informed consent prior to participation. The collected gait data were used to generate normalised reference joint-angle trajectories ($\theta_{\mathrm{ref}}$) for the left and right hip and knee joints under different walking conditions. These trajectories served as nominal target motions for policy learning and controller evaluation. Within the RL environment, system states were updated according to the interaction between the learned control actions and the underlying system dynamics while tracking the reference trajectories.

It should be noted that the healthy gait trajectories employed in this study are not intended to fully represent pathological gait patterns observed in stroke survivors. Rather, they provide nominal reference motions for the initial policy-learning process and controller development. The proposed framework does not assume that patients should exactly reproduce healthy gait patterns. Instead, adaptation to subject-specific conditions is achieved through the integration of biological parameters into the reinforcement learning state and reward spaces, anthropometric domain randomisation during training, and the compliant behaviour of the impedance controller. These mechanisms enable the controller to accommodate inter-subject variability and changing biomechanical conditions. Nevertheless, validation using gait data collected from stroke patients remains necessary to fully assess the clinical generalisability of the proposed approach and constitutes an important direction for future research.

### 3.3. Musculoskeletal Model

**Figure 2:**
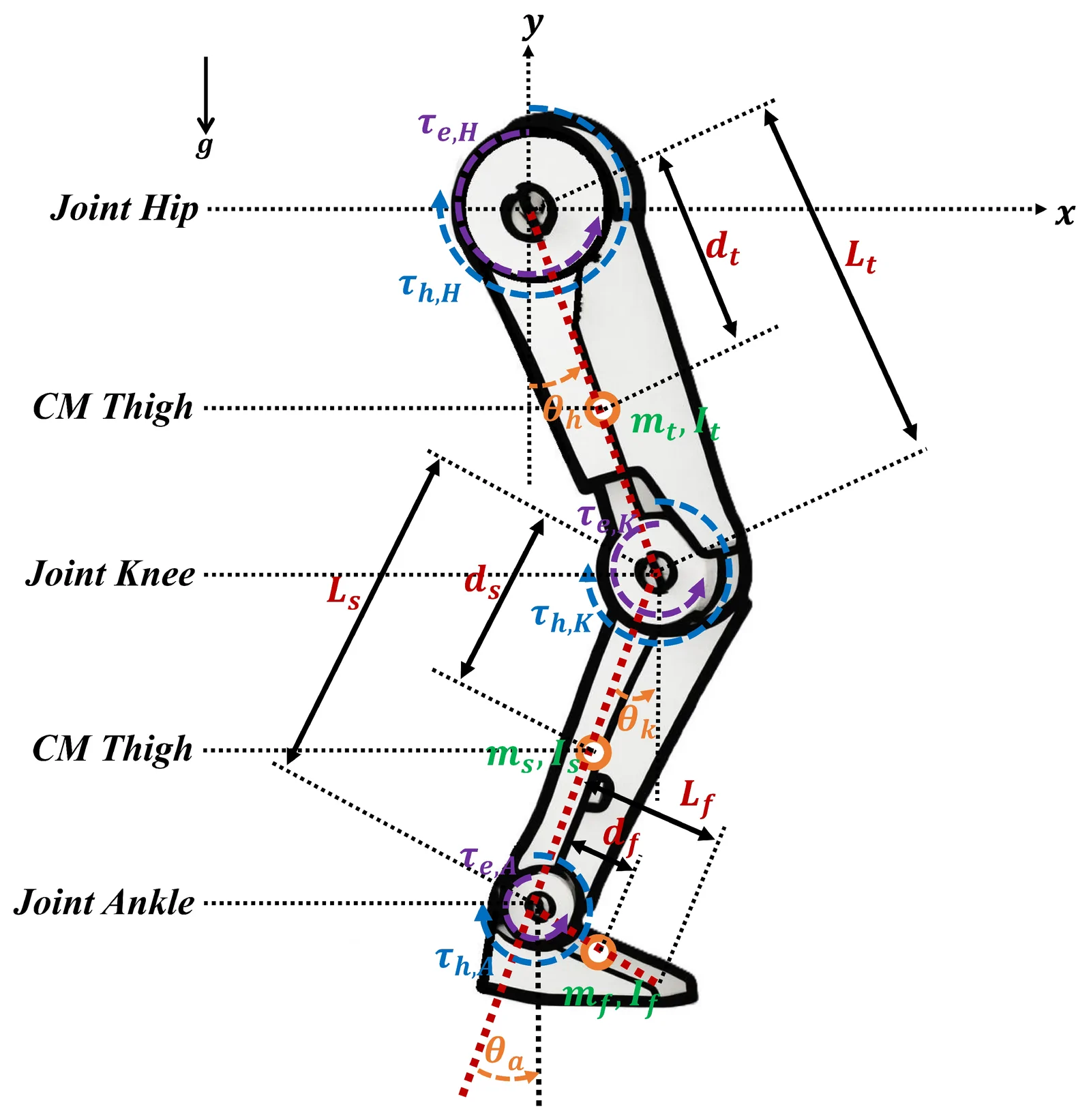
Proposed dynamic model of a two-degree-of-freedom skeletal robot that includes hip, knee, and ankle joints. The centres of mass applied torques (τ) at each joint are specified, along with segment lengths (*L*) and angles (θ). The x and y coordinates, direction of gravity (*g*), and effective masses (*m*) are also shown in the diagram.

Lower-limb exoskeletons typically use mechanical joints and linkages designed to emulate the natural range of motion of human lower limbs. The mechanical design must precisely emulate the kinematic characteristics of human locomotion to provide seamless and natural movement for the user. This section introduces a detailed dynamic model of a two-degrees-of-freedom (2-DoF) lower-limb exoskeleton robot. The model illustrates the relationship between external forces applied to the exoskeleton and the resultant motion of the hip and knee joints. We model each leg as a two-link system (thigh and shank) with hip and knee joints, as seen in Figure 2, using anthropometric parameters derived from average adult human data in Table 1 [18,35].

Given that the principal motion of the lower-limb exoskeleton transpires in the sagittal plane (x−y), the fixed endpoint O(0,0) is designated as the coordinate origin. According to geometric relations, the coordinates of the centre of each link $\left(x_{i},y_{i}\right)$ may be articulated as:

(1) $$\left\{\begin{array}{c} x_{\mathrm{Knee}}=d_{t}\sin\left(\theta_{H}\right)\\ y_{\mathrm{Knee}}=-d_{t}\cos\left(\theta_{H}\right)\\ x_{\mathrm{Ankle}}=d_{t}\sin\left(\theta_{H}\right)+d_{s}\sin\left(\theta_{K}\right)\\ y_{\mathrm{Ankle}}=-d_{t}\cos\left(\theta_{H}\right)-d_{s}\cos\left(\theta_{K}\right) \end{array}\right.$$

where $\theta_{H}$ and $\theta_{K}$ represent the angles at the hip and knee joints. The respective linear velocities of the knee and ankle are derived by differentiating Equation (1):

(2) $$\left\{\begin{array}{c} {\dot{x}}_{\mathrm{Knee}}=d_{t}cos\left(\theta_{H}\right){\dot{\theta }}_{H}\\ {\dot{y}}_{\mathrm{Knee}}=d_{t}sin\left(\theta_{H}\right){\dot{\theta }}_{H}\\ {\dot{x}}_{\mathrm{Ankle}}=d_{t}cos\left(\theta_{H}\right){\dot{\theta }}_{H}+d_{s}cos\left(\theta_{K}\right){\dot{\theta }}_{K}\\ {\dot{y}}_{\mathrm{Ankle}}=d_{t}sin\left(\theta_{H}\right){\dot{\theta }}_{H}+d_{s}sin\left(\theta_{K}\right){\dot{\theta }}_{K} \end{array}\right.$$

The equations of motion are obtained using the Lagrangian approach, defined as:

(3) $$\begin{array}{cc} L= & E_{K,\mathrm{total}}-E_{P,\mathrm{total}}=\left(E_{K,\mathrm{Thigh}}+E_{K,\mathrm{Shank}}\right)-\left(E_{P,\mathrm{Thigh}}+E_{P,\mathrm{Shank}}\right) \end{array}$$

where $E_{K}$ and $E_{P}$ represent the total kinetic and potential energies of the system, respectively. The kinetic and potential energy for each segment are articulated as:

(4) $$E_{K,\mathrm{Thigh}}=\frac{1}{2}m_{t}\left({\dot{x}}_{\mathrm{Knee}}^{2}+{\dot{y}}_{\mathrm{Knee}}^{2}\right)=\frac{1}{2}m_{t}d_{t}^{2}{\dot{\theta }}_{H}^{2},$$

(5) $$E_{K,\mathrm{Shank}}=\frac{1}{2}m_{s}\left({\dot{x}}_{\mathrm{Ankle}}^{2}+{\dot{y}}_{\mathrm{Ankle}}^{2}\right)=\frac{1}{2}m_{s}\left[d_{t}^{2}{\dot{\theta }}_{H}^{2}+d_{s}^{2}{\dot{\theta }}_{K}^{2}+2d_{t}d_{s}{\dot{\theta }}_{H}{\dot{\theta }}_{K}\cos\left(\theta_{H}-\theta_{K}\right)\right],$$

(6) $$E_{P,\mathrm{Thigh}}=m_{t}gy_{\mathrm{Knee}}=-gm_{t}d_{t}\cos\left(\theta_{H}\right),$$

(7) $$E_{P,\mathrm{Shank}}=m_{s}gy_{\mathrm{Ankle}}=-gm_{s}\left(d_{t}\cos\left(\theta_{H}\right)+d_{s}\cos\left(\theta_{K}\right)\right).$$

By substituting Equations (4)–(7) into Equation (3), the Lagrangian function is expressed as:

(8) $$\begin{array}{cc} L= & \frac{1}{2}\left(m_{t}+m_{s}\right)d_{t}^{2}{\dot{\theta }}_{H}^{2}+\frac{1}{2}m_{s}d_{s}^{2}{\dot{\theta }}_{K}^{2}+m_{s}d_{t}d_{s}{\dot{\theta }}_{H}{\dot{\theta }}_{K}\cos\left(\theta_{H}-\theta_{K}\right)\\ \; & +\left(m_{t}+m_{s}\right)gd_{t}\cos\left(\theta_{H}\right)+gm_{s}d_{s}\cos\left(\theta_{K}\right). \end{array}$$

The Euler–Lagrange equation for the *i*-th generalised coordinate is articulated as:

(9) $$\frac{d}{dt}\left(\frac{\partial L}{\partial {\dot{\theta }}_{i}}\right)-\frac{\partial L}{\partial \theta_{i}}=\tau_{s,i}=\tau_{e,i}+\tau_{h,i},$$

where $\tau_{s,i}$ denotes the total torque at the *i*-th joint, including the exoskeleton torque $\tau_{e}$ and the human torque $\tau_{h}$. The Lagrangian equations for the hip and knee joints are formulated as follows:

(10) $$\frac{d}{dt}\left(\frac{\partial L}{\partial {\dot{\theta }}_{H}}\right)-\frac{\partial L}{\partial \theta_{H}}=\tau_{s,H}$$

(11) $$\frac{d}{dt}\left(\frac{\partial L}{\partial {\dot{\theta }}_{K}}\right)-\frac{\partial L}{\partial \theta_{K}}=\tau_{s,K}$$

Derivation of the hip joint:

(12) $$\frac{\partial L}{\partial {\dot{\theta }}_{H}}=\left(m_{t}+m_{s}\right)d_{t}^{2}{\dot{\theta }}_{H}+m_{s}d_{t}d_{s}{\dot{\theta }}_{K}\cos\left(\theta_{H}-\theta_{K}\right),$$

(13) $$\begin{array}{cc} \frac{d}{dt}\left(\frac{\partial L}{\partial {\dot{\theta }}_{H}}\right)= & \left(m_{t}+m_{s}\right)d_{t}^{2}{\ddot{\theta }}_{H}+m_{s}d_{t}d_{s}{\ddot{\theta }}_{K}\cos\left(\theta_{H}-\theta_{K}\right)\\ \; & -m_{s}d_{t}d_{s}{\dot{\theta }}_{K}\sin\left(\theta_{H}-\theta_{K}\right)\left({\dot{\theta }}_{H}-{\dot{\theta }}_{K}\right), \end{array}$$

(14) $$\frac{\partial L}{\partial \theta_{H}}=-\left(m_{t}+m_{s}\right)gd_{t}\sin\left(\theta_{H}\right)-m_{s}d_{t}d_{s}{\dot{\theta }}_{H}{\dot{\theta }}_{K}\sin\left(\theta_{H}-\theta_{K}\right).$$

Substituting Equations (13) and (14) into Equation (10):

(15) $$\begin{array}{cc} \; & \left(m_{t}+m_{s}\right)d_{t}^{2}{\ddot{\theta }}_{H}+m_{s}d_{t}d_{s}\cos\left(\theta_{H}-\theta_{K}\right){\ddot{\theta }}_{K}\\ \; & -m_{s}d_{t}d_{s}\sin\left(\theta_{H}-\theta_{K}\right){\dot{\theta }}_{K}^{2}+\left(m_{t}+m_{s}\right)gd_{t}\sin\left(\theta_{H}\right)=\tau_{s,H}. \end{array}$$

Derivation for the knee joint:

(16) $$\frac{\partial L}{\partial {\dot{\theta }}_{K}}=m_{s}d_{s}^{2}{\dot{\theta }}_{K}+m_{s}d_{t}d_{s}{\dot{\theta }}_{H}\cos\left(\theta_{H}-\theta_{K}\right),$$

(17) $$\begin{array}{cc} \frac{d}{dt}\left(\frac{\partial L}{\partial {\dot{\theta }}_{K}}\right)= & m_{s}d_{s}^{2}{\ddot{\theta }}_{K}+m_{s}d_{t}d_{s}{\ddot{\theta }}_{H}\cos\left(\theta_{H}-\theta_{K}\right)-m_{s}d_{t}d_{s}{\dot{\theta }}_{H}\sin\left(\theta_{H}-\theta_{K}\right)\left({\dot{\theta }}_{H}-{\dot{\theta }}_{K}\right), \end{array}$$

(18) $$\frac{\partial L}{\partial \theta_{K}}=-gm_{s}d_{s}\sin\left(\theta_{K}\right)+m_{s}d_{t}d_{s}{\dot{\theta }}_{H}{\dot{\theta }}_{K}\sin\left(\theta_{H}-\theta_{K}\right).$$

Substituting Equations (17) and (18) into Equation (11) gives:

(19) $$\begin{array}{cc} \; & m_{s}d_{s}^{2}{\ddot{\theta }}_{K}+m_{s}d_{t}d_{s}\cos\left(\theta_{H}-\theta_{K}\right){\ddot{\theta }}_{H}\\ \; & -m_{s}d_{t}d_{s}\sin\left(\theta_{H}-\theta_{K}\right){\dot{\theta }}_{H}^{2}+gm_{s}d_{s}\cos\left(\theta_{K}\right)=\tau_{s,K}. \end{array}$$

The equations may be succinctly represented in conventional matrix form as:

(20) $$M\left(\theta \right)\ddot{\theta }+C\left(\theta ,\dot{\theta }\right)+G\left(\theta \right)=\tau_{s}=\tau_{e}+\tau_{h},$$

where M(θ) denotes the inertia matrix, $C(\theta ,\dot{\theta })$ signifies the coriolis and centrifugal torque vector, and G(θ) indicates the gravitational torque vector.

(21) $$M\left(\theta \right)=\left[\begin{array}{cc} \left(m_{t}+m_{s}\right)d_{t}^{2} & m_{s}d_{t}d_{s}\cos\left(\theta_{H}-\theta_{K}\right)\\ m_{s}d_{t}d_{s}\cos\left(\theta_{H}-\theta_{K}\right) & m_{s}d_{s}^{2} \end{array}\right],$$

(22) $$C\left(\theta ,\dot{\theta }\right)=\left[\begin{array}{c} -m_{s}d_{t}d_{s}\sin\left(\theta_{H}-\theta_{K}\right){\dot{\theta }}_{K}^{2}\\ -m_{s}d_{t}d_{s}\sin\left(\theta_{H}-\theta_{K}\right){\dot{\theta }}_{H}^{2} \end{array}\right],$$

(23) $$G\left(\theta \right)=\left[\begin{array}{c} \left(m_{t}+m_{s}\right)gd_{t}\sin\left(\theta_{H}\right)\\ gm_{s}d_{s}\cos\left(\theta_{K}\right) \end{array}\right].$$

The derived dynamic equations were implemented analytically in MATLAB as separate functions for M(θ), $C(\theta ,\dot{\theta })$, and G(θ). These functions are called at each simulation step within the custom reinforcement learning environment. It should be noted that the current model is a simplified 2-DoF rigid-body dynamic representation per leg and does not include detailed musculoskeletal elements such as individual muscle-tendon units, activation dynamics, or soft-tissue compliance.

### 3.4. Impedance Control

An impedance control approach is used to guarantee compliant and natural mobility in human–robot interaction [35,51,52]. This controller regulates the dynamic interplay between the interaction force and the motion response of the exoskeleton. The control legislation is articulated as follows:

(24) $$\tau_{e}=K\left(\theta_{d}-\theta \right)+D\left({\dot{\theta }}_{d}-\dot{\theta }\right)+\hat{M}\left(\theta \right){\ddot{\theta }}_{d}+\hat{C}\left(\theta ,\dot{\theta }\right)+\hat{G}\left(\theta \right).$$

In this context, $\theta_{d}$, ${\dot{\theta }}_{d}$, and ${\ddot{\theta }}_{d}$ signify the target joint position, velocity, and acceleration, respectively; *K* and *D* refer to the stiffness and damping matrices; and $\hat{M}\left(\cdot \right)$, $\hat{C}\left(\cdot \right)$, $\hat{G}\left(\cdot \right)$ indicate the estimated parameters of the dynamic model. Substituting Equation (24) into Equation (20) yields the closed-loop dynamic equation:

(25) $$K\left(\theta_{d}-\theta \right)+D\left({\dot{\theta }}_{d}-\dot{\theta }\right)+M\left(\theta \right)\left({\ddot{\theta }}_{d}-\ddot{\theta }\right)=\tau_{h}.$$

The tracking error, defined as $e=\theta_{d}-\theta$, may be expressed in terms of error dynamics as follows:

(26) $$Ke+D\dot{e}+M\left(\theta \right)\ddot{e}=\tau_{h},$$

where M(θ) denotes the positive-definite inertia matrix, *D* is the damping matrix, *K* is the stiffness matrix, and $\tau_{h}$ represents external human-interaction disturbances. The numerical values of these parameters are shown in Table 1. The robot functions as a multi-dimensional spring-damper-mass system, dynamically compensating for external torques $\tau_{h}$ (from human contact) to ensure smooth, safe, and natural mobility.

#### Closed-Loop Stability Considerations

The proposed control framework consists of a hierarchical structure comprising a high-level reinforcement learning (RL) policy and a low-level impedance controller. The RL agent generates desired joint trajectories, while the impedance controller is responsible for ensuring stable tracking of the generated references. Considering the tracking error ($e=\theta_{d}-\theta$), the closed-loop error dynamics of the impedance controller can be expressed as Equation (26).

Under the standard assumptions that M(θ) is positive definite and that the impedance matrices *D* and *K* are chosen to be positive definite, the resulting error system behaves as a passive second-order mechanical system. In the absence of external disturbances ($\tau_{h}=0$), the tracking error asymptotically converges toward zero. For bounded disturbances and modelling uncertainties, the closed-loop trajectories remain bounded, owing to the dissipative properties introduced by the damping term.

It should be noted that the RL agent does not directly generate actuator torques. Instead, it provides desired reference trajectories that are subsequently executed by the impedance controller. Therefore, the stability properties of the low-level control loop are preserved while the RL policy serves as a trajectory adaptation mechanism. A rigorous Lyapunov-based stability proof for the complete human–exoskeleton–RL closed-loop system is beyond the scope of the present work and will be investigated in future studies.

### 3.5. Reinforcement Learning Algorithms

Reinforcement learning is a model-free machine learning framework that allows an agent to acquire optimum behaviours by trial-and-error interactions with an environment, hence maximising cumulative rewards over time [53,54]. In reinforcement learning, the issue is articulated as a Markov Decision Process (MDP) characterised by the tuple (S,A,P,R,γ), where S denotes the state space (e.g., joint angles and velocities in the exoskeleton), A represents the action space (e.g., reference torques or angles), $P\left(s^{\prime }|s,a\right)$ indicates the transition probability that delineates state evolution, $R\left(r|s,a\right)$ signifies the reward distribution that offers feedback on actions, and γ∈[0,1) is the discount factor that prioritises future rewards (set to 0.99 for long-term gait optimisation) [55]. The agent aims to identify a policy π that optimises the anticipated return.

(27) $$J\left(\pi \right)=E_{\pi }\;\left[\sum_{t=0}^{\infty }\gamma^{t}r_{t}\right],$$

where $r_{t}$ denotes the immediate reward at time *t*. In the realm of lower-limb exoskeleton control for stroke rehabilitation, RL is especially advantageous due to its capacity to manage continuous state-action spaces (such as real-valued joint angles), adapt to uncertainties (including sensor noise from load cells and variability in patient muscle strength, bone size, or gait patterns), and customise assistance without dependence on explicit dynamic models. This customisation is accomplished by integrating biological characteristics (e.g., scaled muscle mass $m_{t}$ or thigh length $L_{t}$) into the state representation, enabling the agent to adapt policies to specific patients. This study evaluates three deep reinforcement learning algorithms: DDPG, TD3, and SAC. These algorithms were selected for their proven effectiveness in continuous control tasks within robotics [21,42,56,57]. DDPG serves as a foundational deterministic actor-critic method [58], TD3 improves upon it by addressing value overestimation through twin critics and delayed policy updates [59], and SAC incorporates entropy maximisation to enhance exploration in stochastic environments [60].

#### 3.5.1. Agent

In reinforcement learning, the agent is the decision-making entity that engages with the environment by monitoring states $s_{t}\in S$, taking actions $a_{t}\in A$ based on its policy π, obtaining rewards $r_{t}$, and transitioning to subsequent states $s_{t+1}$ [61]. In exoskeleton control, the agent interprets proprioceptive information (e.g., joint angles θ, velocities $\dot{\theta }$, accelerations $\ddot{\theta }$, and torques from load cells) to generate control actions (e.g., reference joint angles or torques for impedance control). The agent architecture employs an actor-critic framework, whereby the actor approximates the policy π (value-free, direct-action selection) and the critic evaluates value functions (Q(s,a) or V(s)), providing a baseline for advantage estimation. This configuration facilitates effective learning in high-dimensional continuous spaces, essential for real-time adaptation to patient-specific dynamics, including variations in muscle mass or bone length included into the simulation environment. The agent’s learning method entails off-policy sampling from a replay buffer $D=(s_{t},\;a_{t},\;r_{t},\;s_{t+1},b$ done) to mitigate temporal correlations and enhance sample efficiency, using target networks for stable updates via Polyak averaging [62].

(28) $$\tau \theta +\left(1-\tau \right)\dot{\theta }\to \dot{\theta },$$

where τ=0.01 denotes the soft update rate. Exploration is harmonised with exploitation: DDPG and TD3 use additive noise ε∼N(0,σ) on actions during training (σmin=0.01), while SAC employs stochastic strategies for intrinsic exploration via entropy. The agent’s performance is assessed using episodic returns, guaranteeing convergence to policies that reduce HEI forces and tracking mistakes.

##### Agent Type

The agents are off-policy actor-critic types, balancing policy optimisation (actor for direct improvement) with value estimation (critic for variance reduction). They replay experiences from the buffer to update parameters asynchronously, enabling high sample efficiency (reuse ratio > 1) [63].

- **DDPG Agent**: A single critic Q(s,a∣ϕ) minimises the temporal difference error via mean squared Bellman error (MSBE):
  (29) $$L\left(\phi \right)=\frac{1}{N}\sum_{i}{\left(y_{i}-Q\left(s_{i},a_{i}|\phi \right)\right)}^{2},$$
  where the target value is
  (30) $$y_{i}=r_{i}+\gamma Q^{\prime }\left(s_{i+1},\mu^{\prime }\left(s_{i+1}|\theta^{\mu^{\prime }}\right)|\phi^{\prime }\right).$$

The actor updates deterministically using the sampled policy gradient. Related sections: target networks prevent divergence (updated softly); Ornstein–Uhlenbeck noise aids exploration in continuous spaces [64].

- **TD3 Agent**: Uses twin critics, $Q_{1}\left(s,a|\phi_{1}\right)$ and $Q_{2}\left(s,a|\phi_{2}\right)$, to compute conservative targets:
  (31) $$y_{i}=r_{i}+\gamma \min\left(Q_{1}^{\prime },Q_{2}^{\prime }\right)\left(s_{i+1},a^{\prime }|\phi_{1}^{\prime },\phi_{2}^{\prime }\right),$$
  with the smooth target action:
  (32) $$a^{\prime }=\mu^{\prime }\left(s^{\prime }|\theta^{\mu^{\prime }}\right)+\varepsilon ,\varepsilon ∼\mathrm{clip}\left(N\left(0,\sigma \right),-c,c\right).$$

The critic losses are:

(33) $$L\left(\phi_{1}\right)=\frac{1}{N}\sum_{i}{\left(y_{i}-Q_{1}\left(s_{i},a_{i}|\phi_{1}\right)\right)}^{2},$$

(34) $$L\left(\phi_{2}\right)=\frac{1}{N}\sum_{i}{\left(y_{i}-Q_{2}\left(s_{i},a_{i}|\phi_{2}\right)\right)}^{2}.$$

Delayed policy updates (every d=2 critic steps) and clipping reduce error propagation and function approximation errors. Ablation studies show improved fault tolerance in noisy environments. Double Q-learning (inspired by Double DQN) mitigates overestimation bias [65,66].

- **SAC Agent**: Employs two critics for soft Q-updates with entropy regularisation. The target value is:
  (35) $$y_{i}=r_{i}+\gamma (\min\left(Q_{1}^{\prime },Q_{2}^{\prime }\right)\left(s_{i+1},a^{\prime }\right)-\alpha \log\pi \left(a^{\prime }|s_{i+1}\right)),a^{\prime }∼\pi \left(\cdot |s_{i+1}\right).$$

The critic loss follows TD3’s form. The state value function is defined as:

(36) $$V\left(s|\psi \right)=E_{a∼\pi }\left[\min\left(Q_{1},Q_{2}\right)\left(s,a\right)-\alpha \log\pi \left(a|s\right)\right],$$

and its loss is:

(37) $$L\left(\psi \right)=\frac{1}{N}\sum_{i}\left(V\left(s_{i}|\psi \right)-E_{a∼\pi }\left[\min\left(Q_{1},Q_{2}\right)\left(s_{i},a\right)-\right.\right.{\left.\left.\alpha \log\pi (a|s_{i})\right]\right)}^{2}.$$

The actor maximises the soft Q-function via reparameterisation. Entropy tuning adapts α dynamically to maintain target entropy, ideal for continuous, high-noise spaces. It draws from maximum entropy RL to achieve an effective exploration–exploitation balance [67].

##### Policy

The policy, represented as π:S→A (deterministic) or π:S→Prob(A) (stochastic), associates states with actions, therefore delineating the agent’s behavioural approach. It is defined by neural network weights $\theta^{\pi }$ and optimised to maximise the aim:

(38) $$J\left(\pi \right)=E_{\pi }\;\left[\sum_{t=0}^{\infty }\gamma^{t}r_{t}\right],$$

where $r_{t}$ is the immediate reward at time *t*, often achieved by gradient climbing on estimated slopes [62].

- In DDPG, the policy is deterministic, $\mu (s|\theta^{\mu })$, and is updated using the deterministic policy gradient theorem:
  (39) $$\nabla_{\theta^{\mu }}J\approx \frac{1}{N}\sum_{i}\left[\nabla_{a}Q\left(s_{i},a_{i}|\phi \right){|}_{a_{i}=\mu \left(s_{i}\right)}\cdot \nabla_{\theta^{\mu }}\mu \left(s_{i}|\theta^{\mu }\right)\right],$$
  where *N* is the mini-batch size, and Q is the critic’s action-value estimate. This encourages actions that maximise Q-values, suitable for precise joint control in exoskeletons, with exploration added as $a_{t}=\mu \left(s_{t}\right)+\varepsilon_{t}$ during training [68].

- In TD3: similar to DDPG, but the target policy is smoothed with clipped noise to regularise:
  (40) $$a^{\prime }=\mu^{\prime }\left(s^{\prime }|\theta^{\mu^{\prime }}\right)+\varepsilon ,\varepsilon ∼\mathrm{clip}\left(N\left(0,\sigma \right),-c,c\right),$$
  where c=0.5, σ=0.2. The policy update is delayed (every d=2 critical steps) to reduce variance and error accumulation:
  (41) $$\nabla_{\theta^{\mu }}J\approx \frac{1}{N}\sum_{i}\left[\nabla_{a}\min\left(Q_{1},Q_{2}\right)\left(s_{i},a_{i}|\phi_{1},\phi_{2}\right)\cdot \nabla_{\theta^{\mu }}\mu \left(s_{i}|\theta^{\mu }\right)\right].$$

This mitigates overestimation bias in Q-values, improving robustness to noisy sensor data like load cell variations [69].

- In SAC: the policy is stochastic, $\pi \left(a|s\right)\approx N(\mu \left(s|\theta^{\mu }\right),\Sigma \left(s|\theta^{\Sigma }\right))$, reparameterised for differentiable sampling:
  (42) $$a=\mu +\Sigma^{1/2}\cdot \xi ,\xi ∼N\left(0,I\right).$$

It maximises the entropy-augmented objective:

(43) $$J\left(\pi \right)=E_{\pi }\left[\sum_{t=0}^{\infty }\left(r_{t}+\alpha H\left(\pi \left(\cdot |s_{t}\right)\right)\right)\right],$$

where

(44) $$H\left(\pi \left(\cdot |s\right)\right)=-E_{a∼\pi }\left[\log\pi (a|s)\right]$$

is the differential entropy term, promoting diverse actions. The policy loss is

(45) $$L_{\pi }\left(\theta^{\pi }\right)=E_{s∼D,\xi ∼N}\left[\alpha \log\pi (f_{\theta^{\pi }}\left(s,\xi \right)|s)\right.\left.-\min\left(Q_{1},Q_{2}\right)\left(s,f_{\theta^{\pi }}\left(s,\xi \right)\right)\right],$$

with α learned via dual optimisation:

(46) $$\nabla_{\alpha }J\left(\alpha \right)=E_{a∼\pi }\left[-\alpha (\log\pi \left(a|s\right)+H_{0})\right],$$

where $H_{0}=-\dim\left(A\right)=-4$ is the target entropy (loge per action dimension for Gaussian policies). This entropy regularisation enhances exploration in uncertain HEI scenarios, allowing adaptation to dynamic patient conditions like varying muscle strengths or gait asymmetries [70].

##### Return

The return, or discounted cumulative reward $G_{t}$, quantifies the long-term value of an action sequence starting from time *t*:

(47) $$G_{t}=\sum_{k=0}^{\infty }\gamma^{k}r_{t+k}=r_{t}+\gamma G_{t+1}.$$

In infinite-horizon MDPs with episodic tasks (e.g., gait cycles), it is estimated via bootstrapping to handle truncation, using value functions for approximation [71].

- **In DDPG/TD3**: returns are approximated by the action-value function:
  (48) $$Q\left(s,a\right)\approx E_{\pi }\left[G_{t}|s_{t}=s,a_{t}=a\right],$$
  bootstrapped as:
  (49) $$y=r+\gamma Q^{\prime }\left(s^{\prime },a^{\prime }\right),$$
  where in TD3 the next action is smoothed for regularisation:
  (50) $$a^{\prime }=\mu^{\prime }\left(s^{\prime }\right)+\varepsilon .$$

The expected trajectory return is computed as:

(51) $$E_{\tau ∼\pi }\left[G_{0}\right]=E\left[\sum_{t=0}^{\infty }\gamma^{t}r_{t}\right].$$

To avoid overestimation bias, TD3 employs conservative targets using twin critics:

(52) $$y=r+\gamma \min(Q_{1}^{\prime },Q_{2}^{\prime }).$$

- **In SAC**: soft returns incorporate entropy regularisation for maximum-entropy RL, modifying the Bellman expectation:
  (53) $$Q\left(s,a\right)\approx E\left[r+\gamma (V\left(s^{\prime }\right)+\alpha H\left(\pi \left(\cdot |s^{\prime }\right)\right))\right],$$
  where the state-value function is defined as:
  (54) $$V\left(s\right)=E_{a∼\pi }\left[Q(s,a)-\alpha \log\pi (a|s)\right].$$

This yields the soft return:

(55) $$G_{t}^{\mathrm{soft}}=\sum_{k=0}^{\infty }\gamma^{k}\left(r_{t+k}+\alpha H\left(\pi \left(\cdot |s_{t+k}\right)\right)\right),$$

which encourages policies that are both high-reward and exploratory. Bootstrapping in SAC uses:

(56) $$y=r+\gamma \left(\min\left(Q_{1}^{\prime },Q_{2}^{\prime }\right)\left(s^{\prime },a^{\prime }\right)-\alpha \log\pi \left(a^{\prime }|s^{\prime }\right)\right),a^{\prime }∼\pi \left(\cdot |s^{\prime }\right).$$

##### Neural Network Architectures

The actor and critic networks used in DDPG, TD3, and SAC were designed to provide consistent feature extraction and value/policy representation for continuous state-action spaces. The selected architectures were determined based on established practices in continuous-control reinforcement learning and preliminary hyperparameter exploration. Several candidate network configurations with different hidden-layer sizes were evaluated during pilot training. Networks with fewer than 128 neurons per hidden layer showed limited capacity to represent the non-linear dynamics of the human–exoskeleton interaction, whereas substantially larger networks increased computational cost, training variance, and convergence time without providing noticeable improvements in tracking accuracy or cumulative reward. Therefore, a three-hidden-layer architecture with 256, 128, and 64 neurons was selected as a compromise between representation capability and computational efficiency. The same hidden-layer configuration was adopted for the corresponding actor networks of DDPG, TD3, and SAC and for the main critic architectures to ensure a fair comparison among the algorithms.

All hidden layers employ Rectified Linear Unit (ReLU) activation functions because of their computational efficiency and effective gradient propagation. For the deterministic actors used in DDPG and TD3, the output layer uses a hyperbolic tangent (tanh) activation function to constrain the four continuous control actions to the normalised range [−1,1]. The SAC actor follows the same hidden-layer architecture but produces the parameters of a stochastic policy, including the action mean and log-standard deviation, with the sampled actions subsequently transformed using tanh to satisfy the bounded action constraints. Network weights are initialised orthogonally and biases are initialised to zero. The Adam optimiser is used for network optimisation, while L2 regularisation with a coefficient of $1\times 10^{-4}$ is applied to improve generalisation and training stability.

**Actor Network:** The actor receives a four-dimensional state vector representing the state variables used by the control system. The network consists of three fully connected hidden layers containing 256, 128, and 64 neurons, respectively, with ReLU activation after each hidden layer. For DDPG and TD3, the final layer contains four neurons with tanh activation, corresponding to the four continuous control actions. In SAC, the same feature-extraction layers are used, while the final policy head parameterises a stochastic distribution over the four-dimensional action space through its mean and log-standard-deviation outputs. The actor parameters are optimised using the Adam optimiser with a learning rate of $10^{-4}$.

**Critic Network:** The critic estimates the action-value function Q(s,a) from both state and action information. For DDPG and TD3, the critic employs a branched architecture in which the four-dimensional state input is processed through fully connected layers of 256 and 128 neurons, while the four-dimensional action input is projected into a 128-neuron layer. The resulting state and action representations are combined and subsequently passed through a 64-neuron ReLU layer, followed by a single linear output neuron that estimates the Q-value. TD3 employs two independent critic networks with the same architecture to reduce overestimation bias through twin-critic learning. DDPG uses a single critic network. For SAC, two independent critic networks are also employed; each processes the combined state-action representation through fully connected layers of 256, 128, and 64 neurons before producing a single scalar Q-value. The critic parameters are optimised using Adam with a learning rate of $10^{-3}$.

The principal neural-network and training hyperparameters used by the three reinforcement learning algorithms are summarised in Table 2. A mini-batch size of 128 and a replay buffer capacity of $10^{6}$ transitions are used for training. The discount factor is set to γ=0.99, while the soft target-network update coefficient is τ=0.01. Training is performed for 500 episodes. These settings are kept consistent across the algorithms wherever applicable to provide a controlled comparison of their learning and control performance.

**Figure 3:**
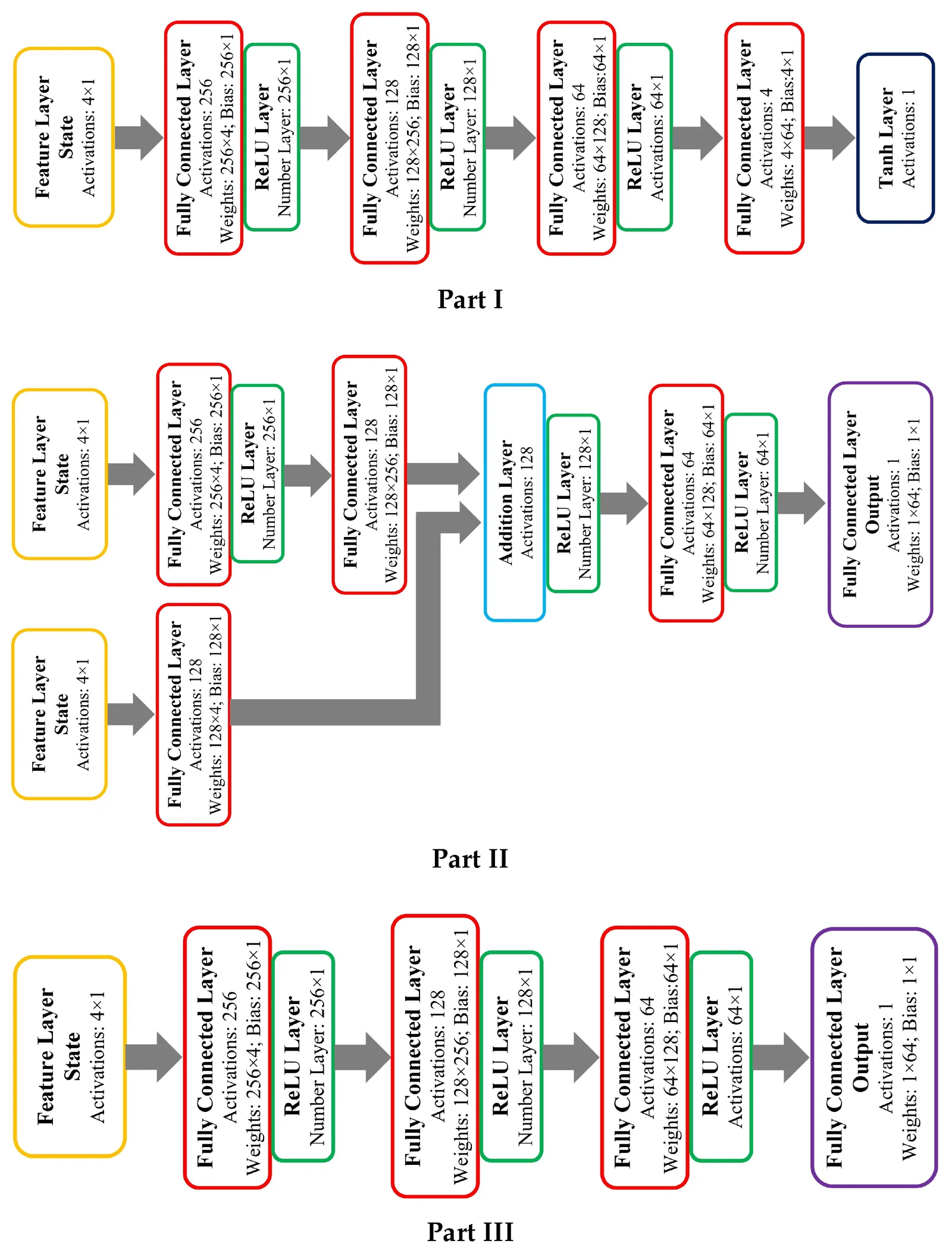
Neural network architectures for the actor and critic components. (**Part I**) Actor network used by DDPG and TD3, with the architecture 4→256→128→64→4 and ReLU activations in the hidden layers followed by tanh activation for bounded actions in [−1,1]. The SAC actor uses the same hidden-layer architecture with a stochastic policy output. (**Part II**) Branched critic architecture used by DDPG and TD3, in which the state pathway (4→256→128) and action pathway (4→128) are combined before the subsequent 64→1 layers. (**Part III**) Critic architecture used by SAC, consisting of the corresponding state-action processing layers followed by 64→1 Q-value estimation. All hidden layers use ReLU activation, and the final critic layer uses a linear activation.

Figure 3 illustrates the neural-network architectures used for the actor and critic components. Part I presents the actor architecture shared by DDPG, TD3, and the feature-extraction component of SAC, consisting of the sequence 4→256→128→64 followed by the corresponding policy output layer. Part II shows the branched critic architecture used by DDPG and TD3, in which the state and action pathways are processed separately before being combined for Q-value estimation. Part III presents the critic architecture used by SAC. All hidden layers use ReLU activation, while the final critic output is linear to produce an unconstrained Q-value. The architectures are designed to provide sufficient non-linear representation capacity for the noisy, patient-specific state information encountered during adaptive exoskeleton control while maintaining computational efficiency suitable for real-time implementation.

#### 3.5.2. Environment of Exoskeleton Robot

The environment simulates the HEI as an MDP, providing observations $o_{t}$ (partial states), rewards $r_{t}$, and transitions $s_{t+1}∼P\left(\cdot |s_{t},a_{t}\right)$. Implemented as rlFunctionEnv in MATLAB, it models bilateral lower limbs with 4 DOF (hip/knee per leg).

##### Simulation Environment

The simulation environment for the lower-limb exoskeleton robot is implemented as an rlFunctionEnv in MATLAB, modelling an MDP with bilateral legs (4 DoF: hip and knee per leg). The environment provides observations, processes actions, computes dynamics, and generates rewards, incorporating noise and randomisation for robustness. Below is the algorithmic representation of the simulation environment, written in pseudocode to detail the step, reset, and termination logic.

The complete simulation pipeline, including the custom environment, impedance controller, and RL agent training, was realised in MATLAB R2025a. Pseudocode for the environment is provided in Algorithm 1. The simulation uses explicit Euler integration with a fixed time step to update joint positions, velocities, and accelerations based on the total torque (dynamics + impedance control). Domain randomisation and additive white Gaussian noise (AWGN) are applied as described in Algorithm 1.

##### Reward Function

The reward function $r_{t}$ guides learning toward optimal exoskeleton control, balancing multiple objectives in a multi-task setup:

(57) $$r_{t}=-0.7e_{\mathrm{track}}-0.5e_{\mathrm{torque}}-0.2s_{\mathrm{smooth}}-0.5I_{\mathrm{bound}},$$

where:

- Mean square error of angles: this term measures the deviation between the desired joint angles ($\theta_{ref}$) and the actual joint angles (θ) at each time step, calculated as following:
  (58) $$\begin{array}{cc} \; & e_{\mathrm{track}}=\frac{1}{4}\sum_{i=1}^{4}{\left(\theta_{i}\left(\mathrm{currentStep}\right)-\theta_{ref,i}\right)}^{2},\\ \; & \theta_{ref,i}=a_{t,i}\frac{\pi }{2}. \end{array}$$

The factor of 1/4 averages the error across the four joints (right hip (RH), right knee (RK), left hip (LH), and left knee (LK)), penalising inaccurate tracking that could lead to unstable gait or misalignment with patient intent. This part ensures that the action output is action with the actual joint angle.

- Mean square error of torques: This term assesses the difference between the computed torque ($\tau_{total}$) and the measured torque from load cells ($\tau_{load}$), computed as following:
  (59) $$e_{\mathrm{torque}}=\frac{1}{4}\sum_{i=1}^{4}\frac{{\left({\mathrm{LoadCell}}_{i}\left(\mathrm{currentStep}\right)-\tau_{\mathrm{total},i}\right)}^{2}}{10}.$$

The division by 10 scales the torque error to a comparable magnitude with other terms, penalising inefficient torque application that might increase energy consumption or strain on actuators.

- Action smoothness: this term quantifies the jerkiness of consecutive actions, calculated as following:
  (60) $$s_{\mathrm{smooth}}=\left\{\begin{array}{cc} \frac{1}{4}\sum_{i=1}^{4}{\left(a_{t,i}-a_{t-1,i}\right)}^{2}, & t>1,\\ 0, & t\leq 1. \end{array}\right.$$

By averaging over the four action dimensions, it penalises abrupt changes in control signals, promoting smooth transitions that enhance patient comfort and reduce mechanical wear.

- Bound violation penalty: it enforces the physical constraint that actions (scaled to [−1,1]) remain within the actuator’s operational limits, preventing unsafe or infeasible commands that could damage the exoskeleton or harm the user, computed as following:
  (61) $$I_{\mathrm{bound}}=\left\{\begin{array}{cc} 1, & \exists i:|a_{t,i}|>1,\\ 0, & \mathrm{otherwise}. \end{array}\right.$$

**Algorithm 1** Simulation of dynamic locomotor system using RL algorithms
  1:**INPUT**: Current step *t*, action $a_{t}$ (4×1 vector [−1,1]), initial state $s_{0}$, gait signal data  2:**OUTPUT**: Next observation $o_{t+1}$, reward $r_{t}$, done flag  3:
**Global Parameters**
  4:M(θ)← inertia matrix [computed via Euler-Lagrange]  5:$C(\theta ,\dot{\theta })\leftarrow$ Coriolis matrix  6:G(θ)← gravity vector  7:$K_{p}\leftarrow \left[500,200\right]$ Nm/rad (hip, knee)  8:$K_{d}\leftarrow \left[50,100\right]$ Nm·s/rad (hip, knee)  9:signal_normalized ← normalized gait data10:SNR ← randomized (5 to ∞ dB)11:randomization_range ←±(15%) (mass, length, inertia)12:
* *
13:**function** Reset()14:    currentStep ←115:    $s_{t}\leftarrow \mathrm{signal}\_\mathrm{normalized}{\left(1,1:4\right)}^{T}$ {Initial joint angles}16:    ${\dot{\theta }}_{t}\leftarrow \frac{\nabla (\mathrm{signal}\_\mathrm{normalized}(1:3,1:4))}{dt}$ {Velocity via central difference}17:    ${\ddot{\theta }}_{t}\leftarrow \frac{\nabla ({\dot{\theta }}_{t}\left(1:2,:\right))}{dt}$ {Acceleration}18:    biological_params $\leftarrow [m_{t}\left(1+\mathrm{rand}\left(-0.15,0.15\right)\right),L_{t}\left(1+\mathrm{rand}\left(-0.15,0.15\right)\right)]$ {Randomized}19:    $o_{t}\leftarrow \left[s_{t};{\dot{\theta }}_{t};{\ddot{\theta }}_{t};\mathrm{biological}\_\mathrm{params}\right]+\mathrm{AWGN}\left(s_{t},\mathrm{SNR}\right)$ {Add noise}20:    loggedSignals $\leftarrow [o_{t}]$21:    **return** $o_{t}$22:
**end function**
23:
* *
24:**function** Step($a_{t}$)25:    {Extract current state from logged signals}26:    $s_{t}\leftarrow \mathrm{loggedSignals}(\mathrm{end},\;1:4)$ {Current joint angles}27:    ${\dot{\theta }}_{t}\leftarrow \mathrm{loggedSignals}(\mathrm{end},\;5:8)$ {Current velocities}28:    ${\ddot{\theta }}_{t}\leftarrow \mathrm{loggedSignals}(\mathrm{end},\;9:12)$ {Current accelerations}29:    biological_params ←loggedSignals(end,13:14) {Current bio params}30:    {Compute dynamics with randomized parameters}31:    $M_{\mathrm{rand}}\leftarrow M\left(\theta_{t}\right)\left(1+\mathrm{rand}\left(-0.15,0.15\right)\right)$ {Randomize inertia}32:    $C_{\mathrm{rand}}\leftarrow C\left(\theta_{t},{\dot{\theta }}_{t}\right)\left(1+\mathrm{rand}\left(-0.15,0.15\right)\right)$33:    $G_{\mathrm{rand}}\leftarrow G\left(\theta_{t}\right)\left(1+\mathrm{rand}\left(-0.15,0.15\right)\right)$34:    $\tau_{\mathrm{dynamics}}\leftarrow M_{\mathrm{rand}}{\ddot{\theta }}_{t}+C_{\mathrm{rand}}{\dot{\theta }}_{t}+G_{\mathrm{rand}}$ {Inverse dynamics}35:    {Apply impedance control with action as reference}36:    $\theta_{\mathrm{ref}}\leftarrow a_{t}\frac{\pi }{2}$ {Scale action to angle range $\left[-\frac{\pi }{2},\frac{\pi }{2}\right]$}37:    ${\dot{\theta }}_{\mathrm{ref}}\leftarrow \frac{\nabla (\theta_{\mathrm{ref}})}{dt}$ {Reference velocity}38:    $\tau_{\mathrm{imp}}\leftarrow K_{p}⊙\left(\theta_{\mathrm{ref}}-s_{t}\right)+K_{d}⊙\left({\dot{\theta }}_{\mathrm{ref}}-{\dot{\theta }}_{t}\right)$ {Impedance torque}39:    {Total torque and update state}40:    $\tau_{\mathrm{total}}\leftarrow \tau_{\mathrm{dynamics}}+\tau_{\mathrm{imp}}$41:    ${\ddot{\theta }}_{t+1}\leftarrow M_{\mathrm{rand}}^{-1}\left(\tau_{\mathrm{total}}-C_{\mathrm{rand}}{\dot{\theta }}_{t}-G_{\mathrm{rand}}\right)$ {Acceleration update}42:    ${\dot{\theta }}_{t+1}\leftarrow {\dot{\theta }}_{t}+{\ddot{\theta }}_{t+1}dt$ {Velocity update}43:    $s_{t+1}\leftarrow s_{t}+{\dot{\theta }}_{t+1}dt$ {Position update}44:    {Add noise to next state}45:    noise $\leftarrow \sqrt{\frac{\mathrm{var}(s_{t+1})}{10^{\mathrm{SNR}/10}}}\times \mathrm{randn}\left(\mathrm{size}\left(s_{t+1}\right)\right)$ {AWGN}46:    $o_{t+1}\leftarrow \left[s_{t+1};{\dot{\theta }}_{t+1};{\ddot{\theta }}_{t+1};\mathrm{biological}\_\mathrm{params}\right]+\mathrm{noise}$47:    {Update logged signals}48:    loggedSignals $\leftarrow [\mathrm{loggedSignals};o_{t+1}]$49:    {Check termination}50:    done ←(currentStep≥length(signal_normalized)) OR $\left(\max(\right|s_{t+1}\left|)>\pi \right)$ OR $\left(e_{\mathrm{track}}>10\right)$51:    **if** done **then**52:        currentStep ←1 {Reset for next episode}53:    **else**54:        currentStep ← currentStep +155:    **end if**56:    **return** $o_{t+1}$, $r_{t}$, done57:
**end function**
58:
* *
59:**function** AWGN(signal, SNR)60:    signal_power ← var(signal)61:    noise_power $\leftarrow \frac{\mathrm{signal}\_\mathrm{power}}{10^{\mathrm{SNR}/10}}$62:    **return** $\mathrm{randn}(\mathrm{size}(\mathrm{signal}))\sqrt{\mathrm{noise}\_\mathrm{power}}$63:
**end function**
64:
* *
65:**function** gradient(signal_window)66:    **return** $\nabla \left(\mathrm{signal}\_\mathrm{window}\right)=\frac{\mathrm{signal}\_\mathrm{window}(\mathrm{end})-\mathrm{signal}\_\mathrm{window}(1)}{\left(\mathrm{length}(\mathrm{signal}\_\mathrm{window})dt\right)}$67:
**end function**



For initial steps (t≤3): $r_{t}=-0.7e_{\mathrm{track}}$ to bootstrap learning without derivatives. Rewards are scalar, real-valued, bounded [−10,0] for stability, and computed per step to encourage sparse, shaped feedback. This design promotes accurate tracking, energy-efficient torques, smooth motions, and adherence to physical limits while handling uncertainties through negative weighting.

The coefficients in the reward function (0.7 for $e_{\mathrm{track}}$, 0.5 for $e_{\mathrm{torque}}$, 0.2 for $s_{\mathrm{smooth}}$, and 0.5 for $I_{\mathrm{bound}}$) were determined empirically through preliminary training runs with TD3. The dominant weight on tracking error reflects the primary goal of gait replication in rehabilitation, while the remaining terms promote energy-efficient torque application, smooth motion for patient comfort, and safety by penalising bound violations. The objective was to establish a practical balance among multiple rehabilitation requirements, including trajectory tracking accuracy, torque efficiency, motion smoothness, and safety-related constraints. Different coefficient combinations were evaluated during pilot training runs, and the final values were selected based on their ability to improve learning stability and overall control performance. Although the adopted coefficients produced satisfactory results across all evaluated scenarios, they do not represent a mathematically optimal solution.

### 3.6. Experimental Setup and Evaluation Metrics

The experimental setup and evaluation framework are designed to rigorously assess the performance of the proposed RL algorithms, DDPG, TD3, and SAC for controlling a lower-limb exoskeleton in stroke rehabilitation. The evaluation framework is entirely simulation-based and uses real human gait data as reference inputs.

#### 3.6.1. Experimental Setup

The reinforcement learning algorithms are executed and taught via the MATLAB 2025a RL Toolbox. The computational platform comprises a 13-core Intel Core i7 processor operating at 3.4 GHz, 16 GB of RAM, and an NVIDIA GeForce RTX 3060 Ti GPU, offering enough computing capacity for deep neural network training and dynamic simulations. The training settings are established as follows: a learning rate of $1\times 10^{-4}$ for actors and $1\times 10^{-3}$ for critics, a discount factor γ of 0.99, an experience replay buffer size of $1\times 10^{6}$, a mini-batch size of 128, and Ornstein–Uhlenbeck noise with a standard deviation of 0.3 and a decay rate of $1\times 10^{-5}$ for exploration in DDPG and TD3. SAC integrates an entropy coefficient α with automated adjustment aimed at $H_{0}=-4$ (log(e) per action dimension), refined using dual gradient ascent optimisation. The training consists of 500 episodes, each lasting up to 200 steps of the normalised gait signal, concluding when the average reward surpasses −0.1. Domain randomisation is implemented by altering mass ($m_{t}$ ± 15%), length ($L_{t}$ ± 15%), and signal-to-noise ratio (SNR: 5-∞ dB) to replicate biological fluctuations (e.g., muscular weakness in hemiplegic patients) and environmental uncertainties (e.g., uneven terrain).

#### 3.6.2. Evaluation Metrics

Performance is evaluated using a comprehensive set of metrics derived from training statistics and post-training simulations, analysed via custom MATLAB scripts. The evaluation encompasses tracking accuracy, torque efficiency, motion smoothness, robustness to noise, and statistical properties of the learned policies. The following metrics are computed:

- **Reward-based metrics**: These include mean reward (average performance), standard deviation of rewards (consistency), maximum and minimum rewards (range of performance), final mean reward (last 20% of episodes), final standard deviation (stability in convergence), cumulative reward (total performance), normalised cumulative reward (per-episode efficiency), learning improvement (difference between second and first half means), and learning ratio (relative improvement). For example, a positive learning improvement indicates progressive learning, as observed in SAC’s entropy-driven exploration.
- **Tracking error**: Quantified as the mean absolute error (MAE), root mean square error (RMSE), and standard deviation of position errors between real positions and estimated positions. They are calculated as follows:
  (62) $$\mathrm{MAE}=\frac{1}{n}\sum |p_{\mathrm{real}}-p_{\mathrm{estimated}}|,$$
  (63) $$\mathrm{RMSE}=\sqrt{\frac{1}{n}\sum {\left(p_{\mathrm{real}}-p_{\mathrm{estimated}}\right)}^{2}}.$$

## 4. Results

### 4.1. Training Performance Analysis

**Figure 4:**
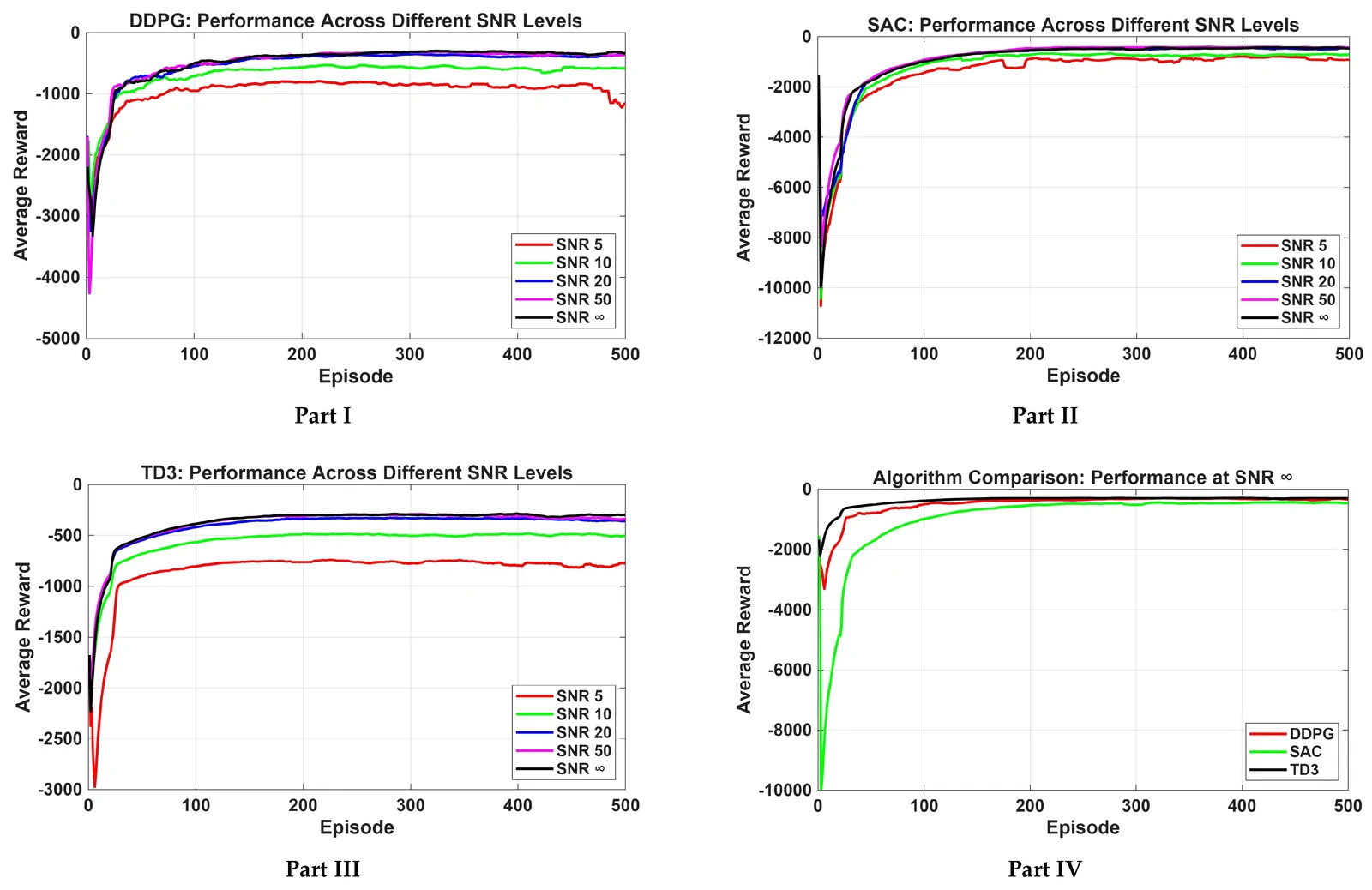
Learning curves of DDPG in Part I, SAC in Part II, and TD3 in Part III algorithms across different SNRs (5, 10, 20, 50, and ∞ dB), and comparison of the three algorithms under noise-free conditions in Part IV. The plots display episode rewards over 500 training episodes, with each curve representing the average reward trend for a specific algorithm and SNR condition. The x-axis denotes episode number, and the y-axis shows reward values, highlighting convergence rates and stability. TD3 exhibits the steadiest ascent, particularly at higher SNR, while SAC shows significant improvement despite initial fluctuations, and DDPG demonstrates moderate stability with noise reduction.

The training performance of the three deep reinforcement learning algorithms (DDPG, TD3, and SAC) was evaluated over 500 episodes in a continuous action space under varying signal-to-noise ratios (SNR = 5, 10, 20, 50, and ∞ dB). Domain randomisation was applied to biological parameters (mass and length) to simulate patient-specific variability. The learning curves for DDPG, SAC, and TD3 across different SNR levels are presented in Figure 4, while the quantitative performance metrics are summarised in Table 3. A more complete table is provided where the performance of the algorithms at different SNR levels (5, 10, 20, 50, and ∞ dB) is evaluated in Appendix A Table A1 to fully evaluate their effectiveness in controlling the lower-limb exoskeleton for stroke rehabilitation.

As shown in Figure 4 and Table 3, TD3 consistently demonstrated the best overall stability and final performance in noise-free conditions. At SNR = ∞ dB, TD3 achieved the highest mean reward of −354.24±18.7, outperforming DDPG (−368.1±22.5) and SAC (−372.5±25.3). TD3 also exhibited smoother convergence and lower variance throughout training, indicating superior stability for exoskeleton control tasks.

DDPG showed competitive performance, particularly in terms of tracking accuracy. It achieved the lowest angle RMSE of 0.0369±0.004rad at SNR = ∞ dB, highlighting its strength in precise joint-angle tracking. However, its performance degraded more noticeably under high noise compared to TD3. SAC demonstrated remarkable robustness in noisy environments. At SNR = 5 dB, SAC obtained the highest learning ratio (0.51), indicating strong adaptive capability and effective exploration even under significant sensor noise. This characteristic makes SAC particularly suitable for real-world scenarios where sensor signals are prone to disturbance. Table 3 presents a detailed comparison at the extreme conditions (SNR = ∞ dB and 5 dB).

The detailed per-SNR performance metrics (extended table in Appendix A) further confirm that TD3 maintains superior final reward and lower variance across most noise levels, while SAC exhibits better relative improvement (learning ratio) in highly noisy conditions. Learning curves in Figure 4 visually corroborate these findings; TD3 displays the steadiest and fastest convergence, particularly at higher SNRs, whereas SAC shows more initial fluctuation but strong recovery.

### 4.2. Angle Estimation Performance

**Figure 5:**
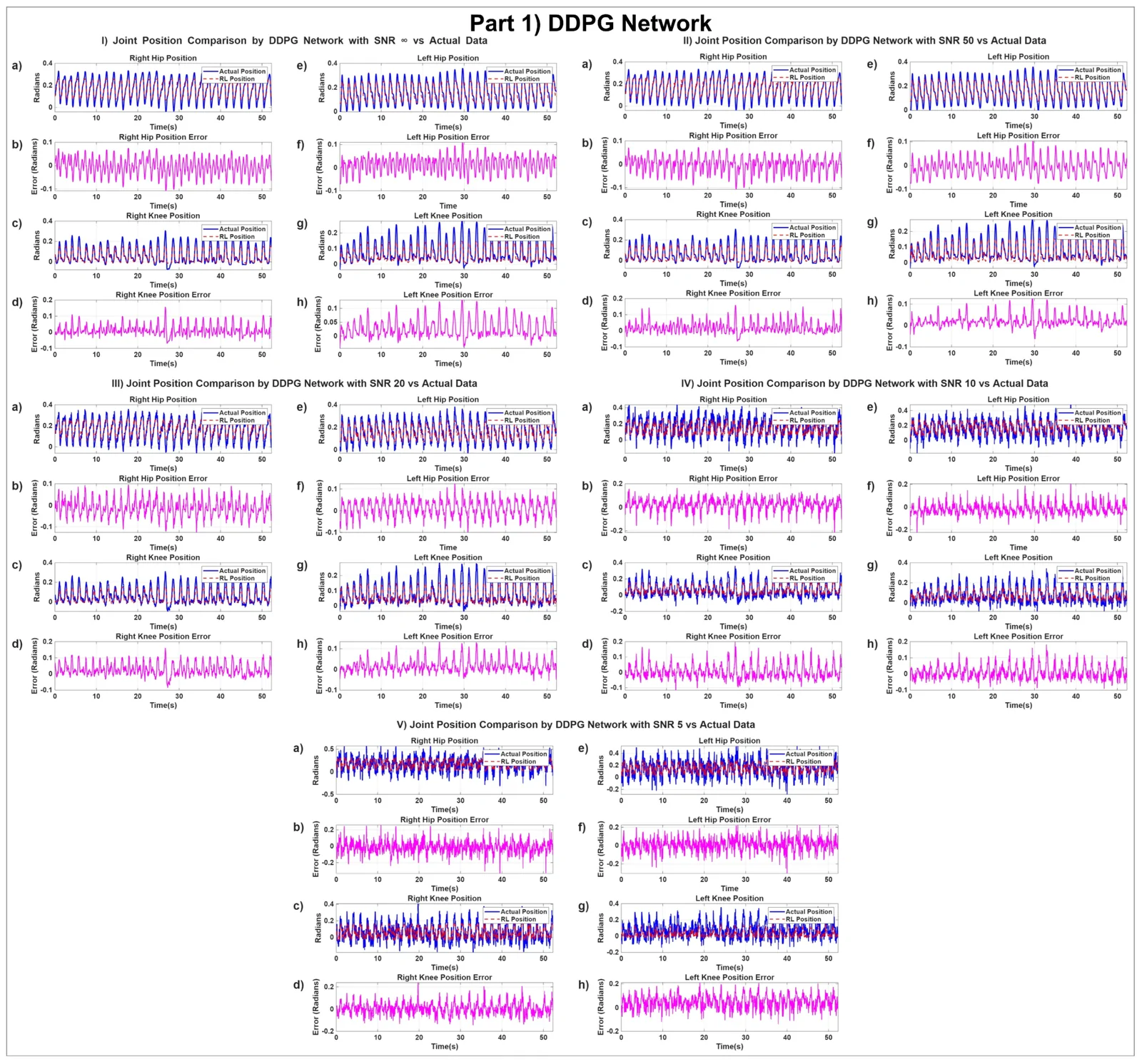

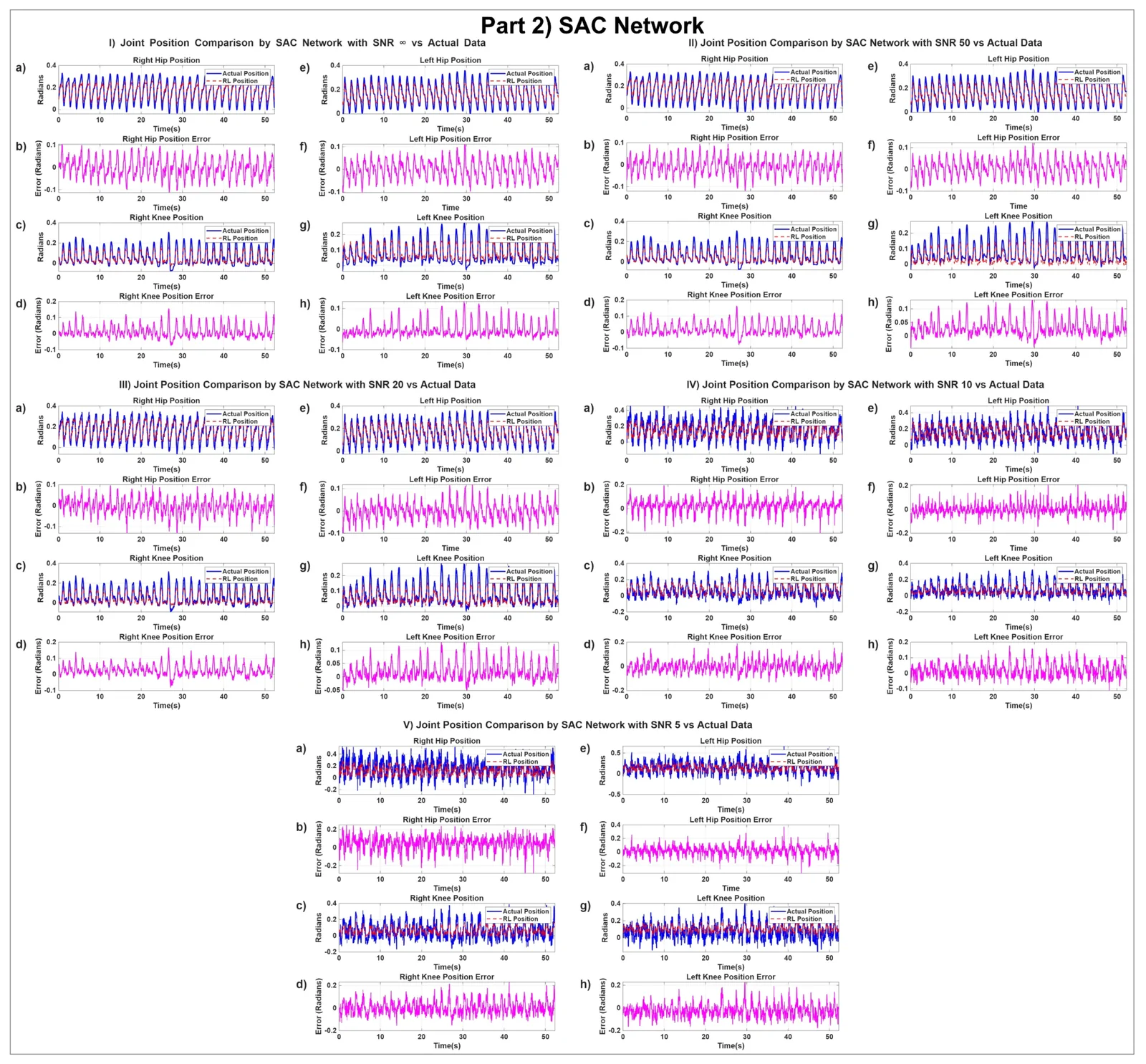

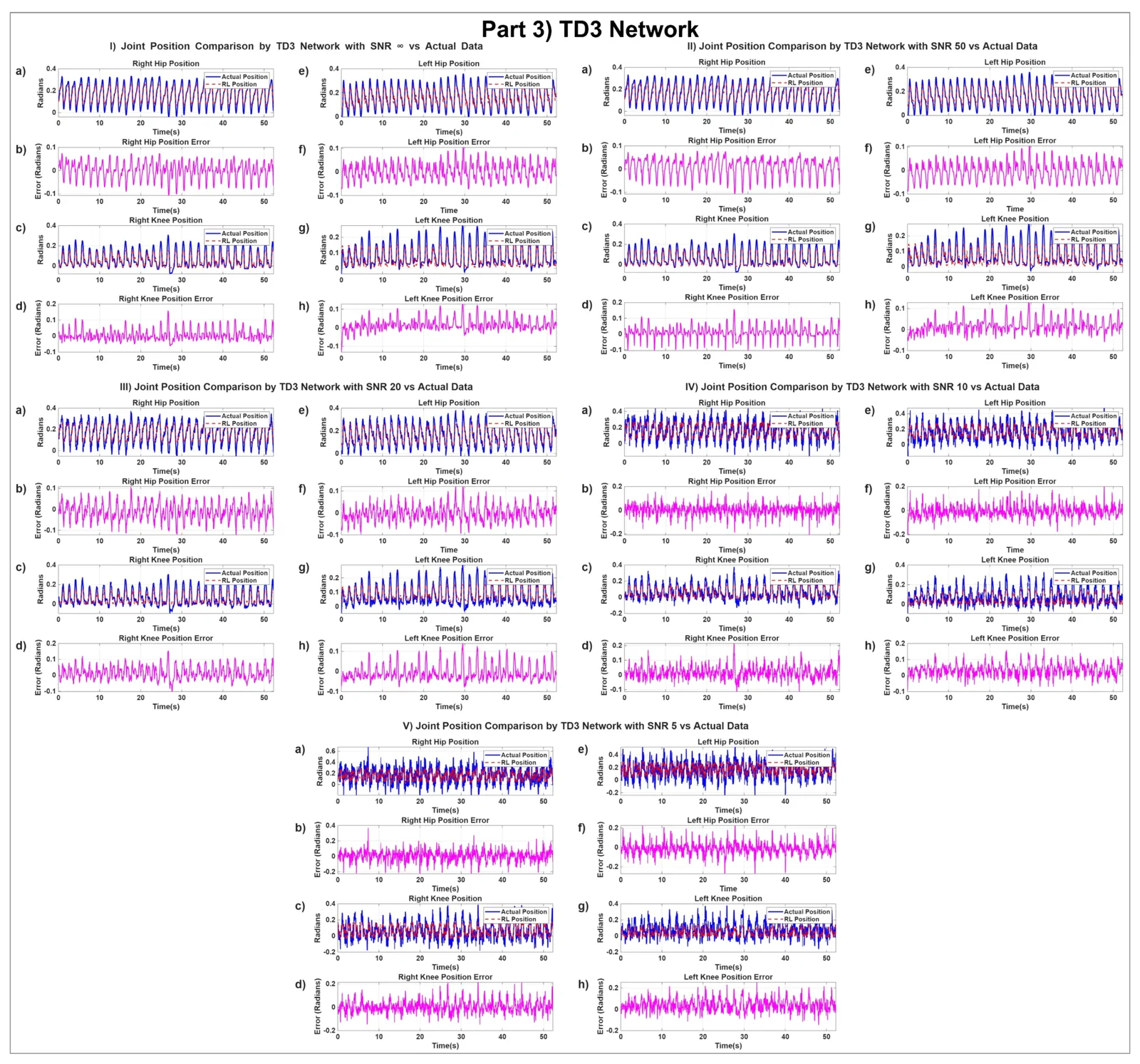
Comparison of predicted joint positions by DDPG, SAC, and TD3 networks with real data at different SNRs. The responses of DDPG, SAC, and TD3 networks in parts (1), (2), and (3) for reconstructing the hip and knee joint positions of the right and left legs under different noise levels (SNR = 5, 10, 20, 50, and ∞ dB at V, IV, III, II, I) are compared with real data. Plots (**a**,**c**,**e**,**g**) show the predicted and real joint positions, while plots (**b**,**d**,**f**,**h**) show the position reconstruction error at each joint over time. Increasing noise increases the error fluctuations and reduces the accuracy of the position estimation at the network output.

To evaluate the tracking accuracy of the learned policies, the predicted joint angles (RH, RK, LH, LK) generated by each RL algorithm were compared with the reference human gait data. The comparison was performed under different noise levels (SNR = 5, 10, 20, 50, and ∞ dB). Figure 5 illustrates the joint position tracking and corresponding errors for all four algorithms, while metrics including mean error (ME), standard error deviation (EST), RMSE, MAE, *p*-value from statistical tests (indicating non-normality), and correlation coefficient (CC) between actual and estimated angles from post-training simulations are presented in Appendix A Table A2. As shown in Figure 5 (Parts 1–3), TD3 and DDPG demonstrated the best visual tracking performance, closely following the reference trajectories even under moderate noise. In contrast, SAC exhibited slightly larger fluctuations, particularly at lower SNRs.

At SNR = ∞ dB (noise-free condition), DDPG achieved the best overall angle tracking accuracy with the lowest RMSE of 0.0369rad across most joints. TD3 showed very competitive performance with RMSE values around 0.035–0.0365rad, demonstrating excellent stability. SAC had slightly higher errors (RMSE ≈0.038–0.042rad) but maintained strong correlation coefficients (>0.93). Under noisy conditions (SNR = 5 dB), all algorithms experienced performance degradation, as expected, TD3 maintained the most robust performance with the lowest RMSE values among the three successful algorithms (approximately 0.050–0.070rad). DDPG showed good accuracy at higher SNRs but degraded more rapidly in high-noise scenarios. SAC demonstrated reasonable robustness, particularly in maintaining acceptable correlation coefficients. Error fluctuations increased significantly with decreasing SNR, as clearly visible in the error plots of Figure 5.

The results confirm that the proposed RL-based controllers (particularly TD3 and DDPG) can accurately replicate human gait trajectories with high precision in simulation. The incorporation of patient-specific biological parameters and domain randomisation enabled the agents to maintain acceptable performance even under noisy sensor conditions, which is critical for real-world deployment in stroke rehabilitation.

### 4.3. Torque Estimation Performance

**Figure 6:**
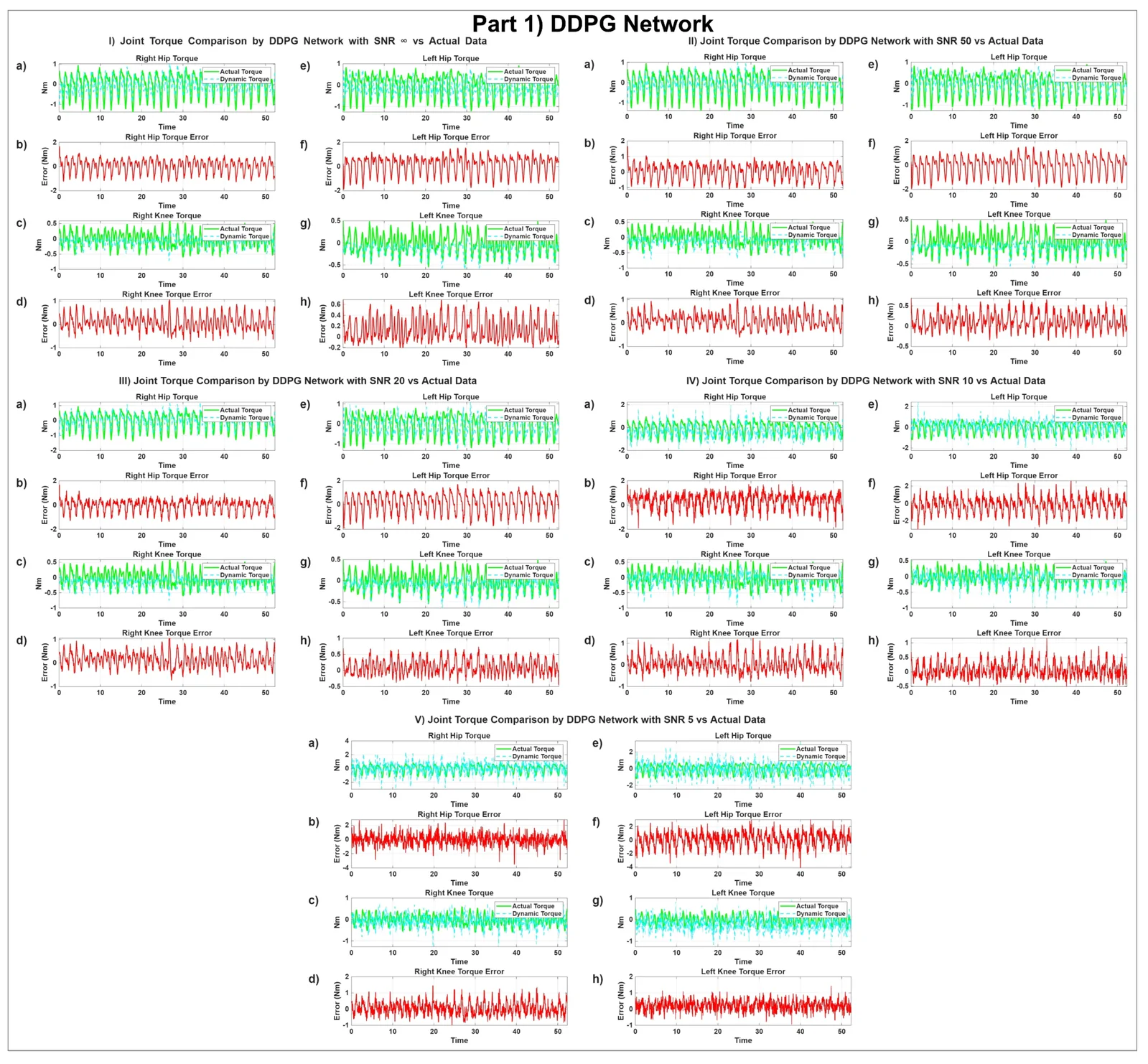

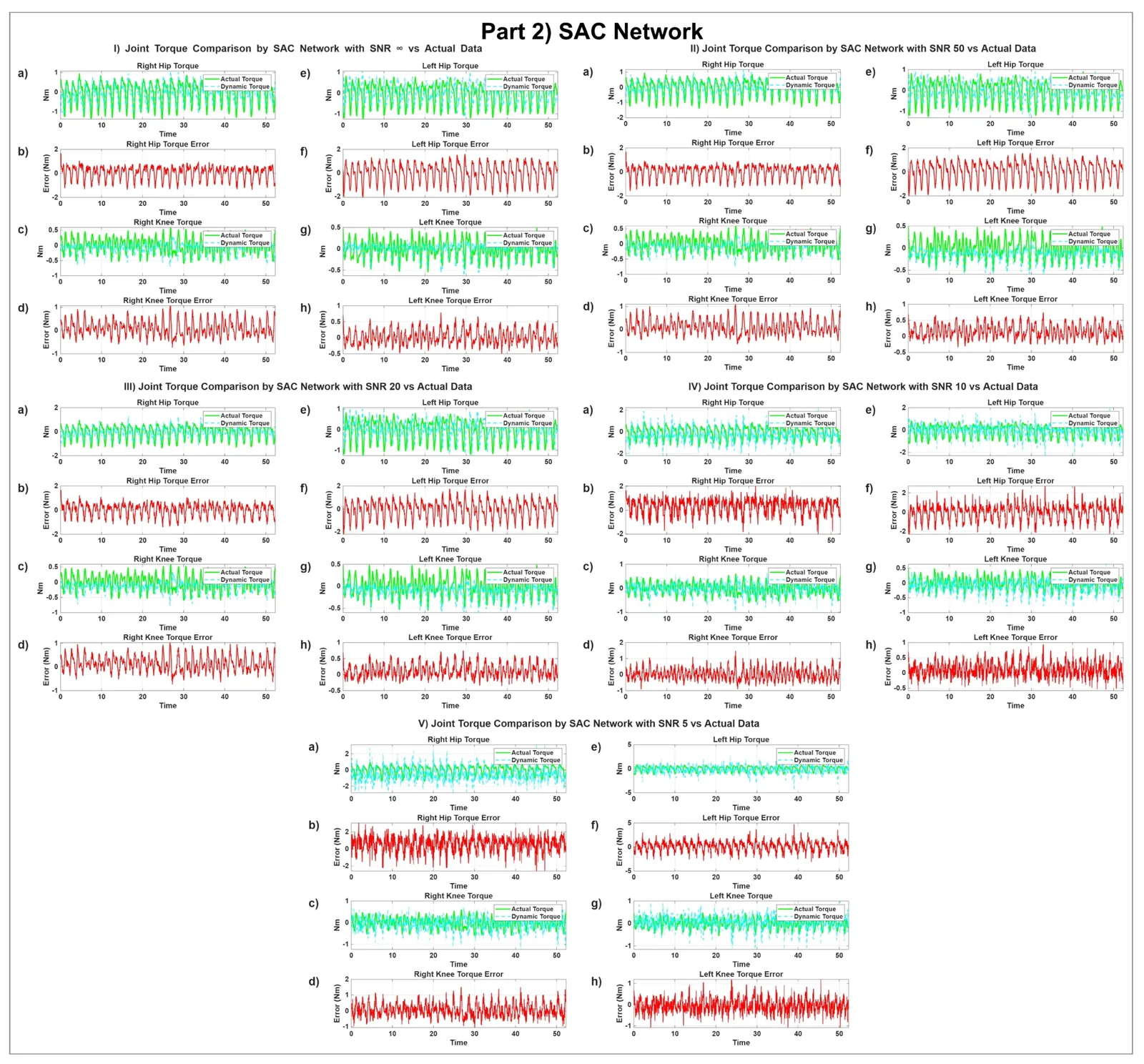

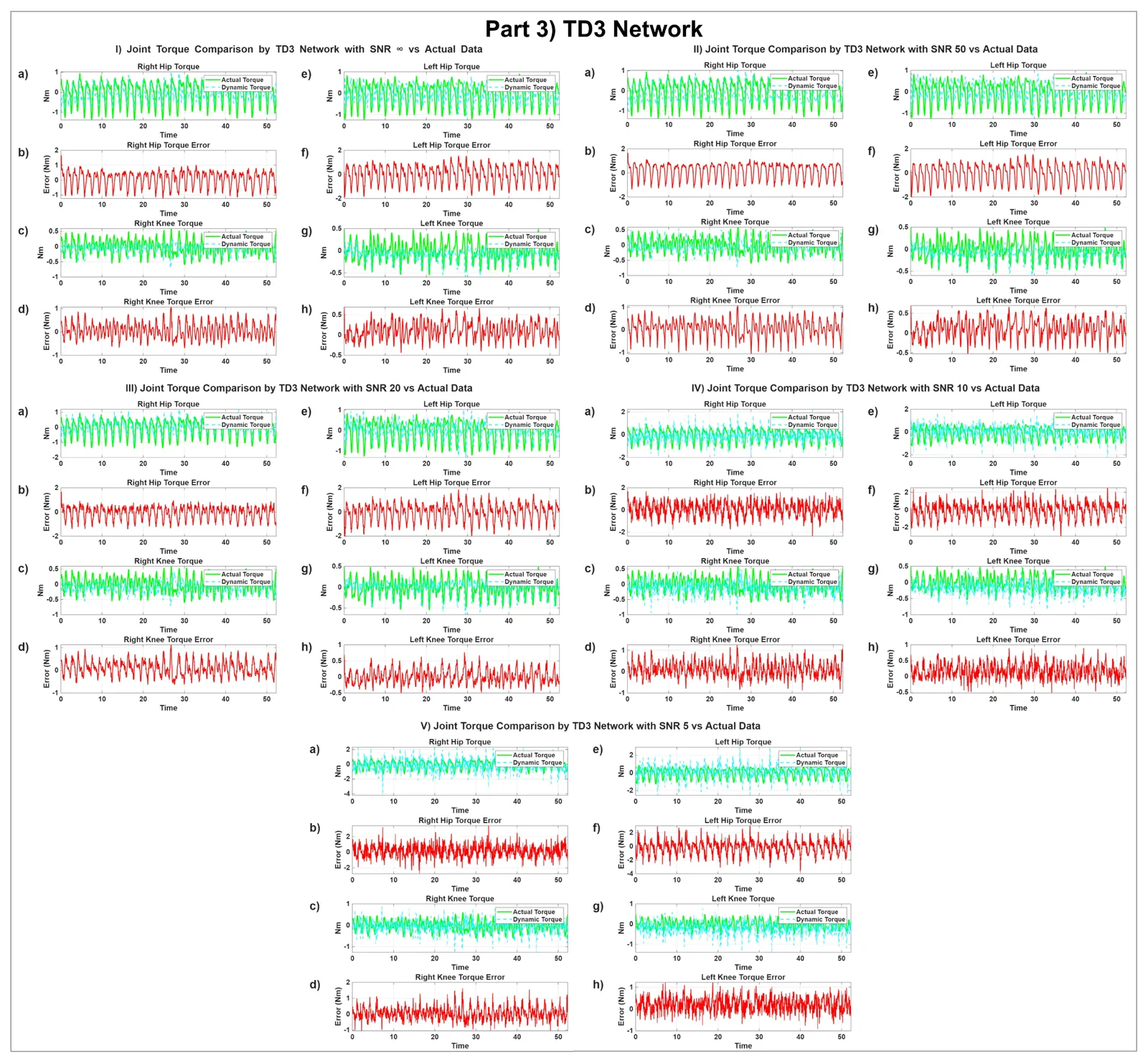
Comparison of joint torques reconstructed by DDPG, SAC, and TD3 networks with real data at different SNRs. The response of (1) DDPG, (2) SAC, and (3) TD3 networks for reconstructing hip and knee joint torques in the right and left legs under different noise levels (SNR = 5, 10, 20, 50, and ∞ dB in V, IV, III, II, I) is compared with real data. Plots (**a**,**c**,**e**,**g**) show the estimated and actual joint torques, while plots (**b**,**d**,**f**,**h**) show the torque reconstruction error at each joint over time. Increasing noise increases the error fluctuations and reduces the accuracy of the torque estimation at the network output.

Accurate torque estimation is crucial for safe and efficient human–exoskeleton interaction, as it directly affects actuator commands and patient comfort. The torque estimation capability of the trained RL policies (DDPG, SAC, and TD3) was evaluated by comparing the predicted joint torques with the reference dynamic torques across the four joints (RH, RK, LH, LK) under different SNR levels. The results are visualised in Figure 6, and metrics including ME, EST, RMSE, MAE, and *p*-value from statistical tests (indicating non-normality) and CC between actual and estimated angles from post-training simulations are presented in Appendix A Table A3. As illustrated in Figure 6 (Parts 1–3), TD3 and DDPG produced torque profiles that most closely followed the reference signals, particularly at higher SNRs. SAC showed acceptable tracking but with noticeably larger fluctuations.

At SNR = ∞ dB (ideal condition), TD3 achieved the best torque estimation performance with the lowest RMSE values (approximately 0.521–0.736Nm across joints). DDPG and SAC showed comparable but slightly higher errors. All three off-policy algorithms maintained reasonable correlation coefficients, although torque prediction proved more challenging than angle tracking (CC typically between 0.29 and 0.45). Under high noise conditions (SNR = 5 dB), torque estimation errors increased significantly for all algorithms. DDPG recorded the highest RMSE at this level (1.0591Nm for one joint), indicating greater sensitivity to noise in torque prediction. TD3 maintained relatively better robustness with lower error growth compared to the others. SAC showed moderate performance but higher variability. Mean errors remained relatively small for successful algorithms, but error standard deviations increased markedly with noise.

These findings align with the training performance results, reinforcing that TD3 offers superior overall performance for adaptive and robust control of lower-limb exoskeletons in noisy and uncertain environments.

### 4.4. Advanced Error Distribution Analysis

**Figure A1:**
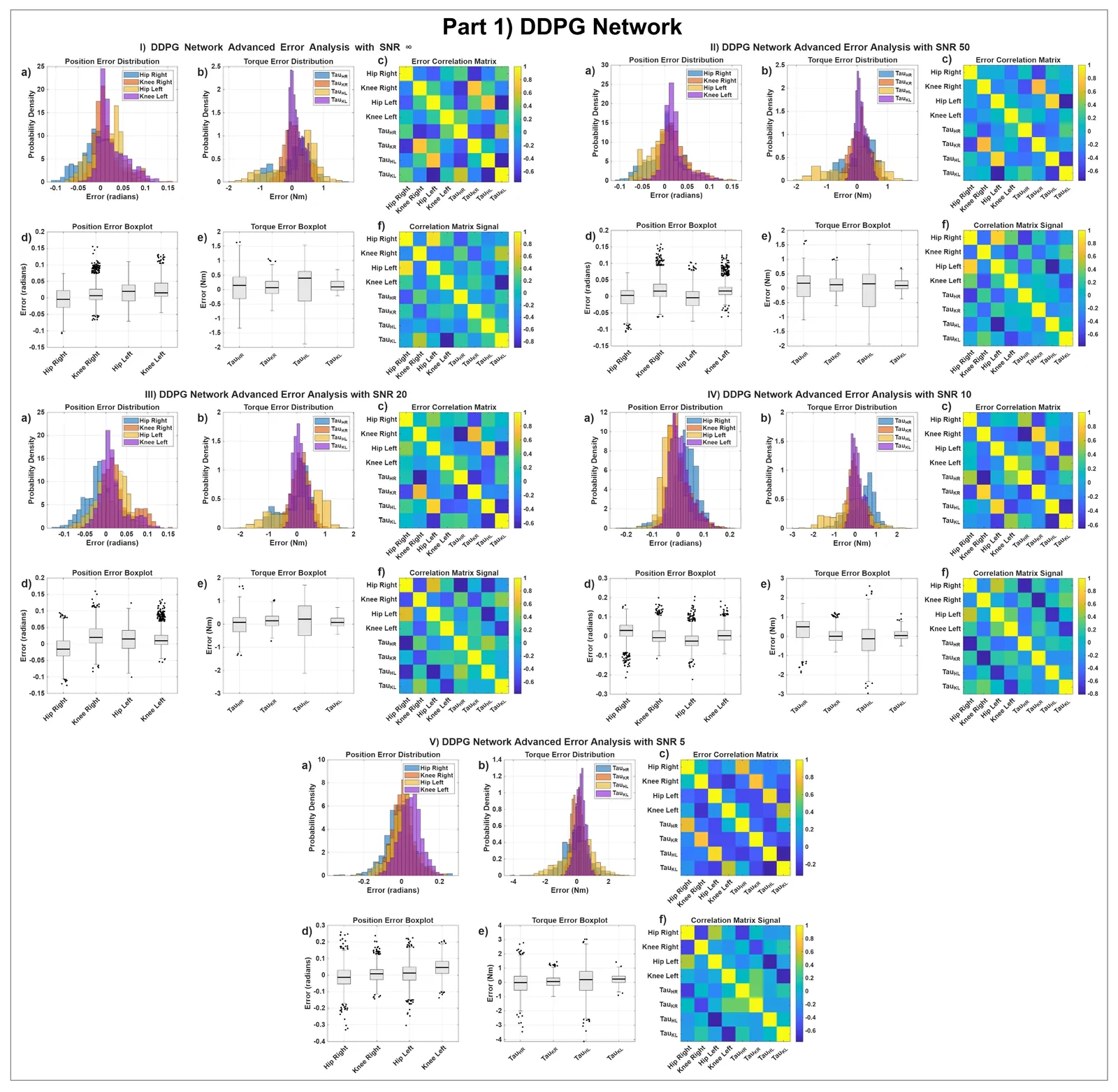

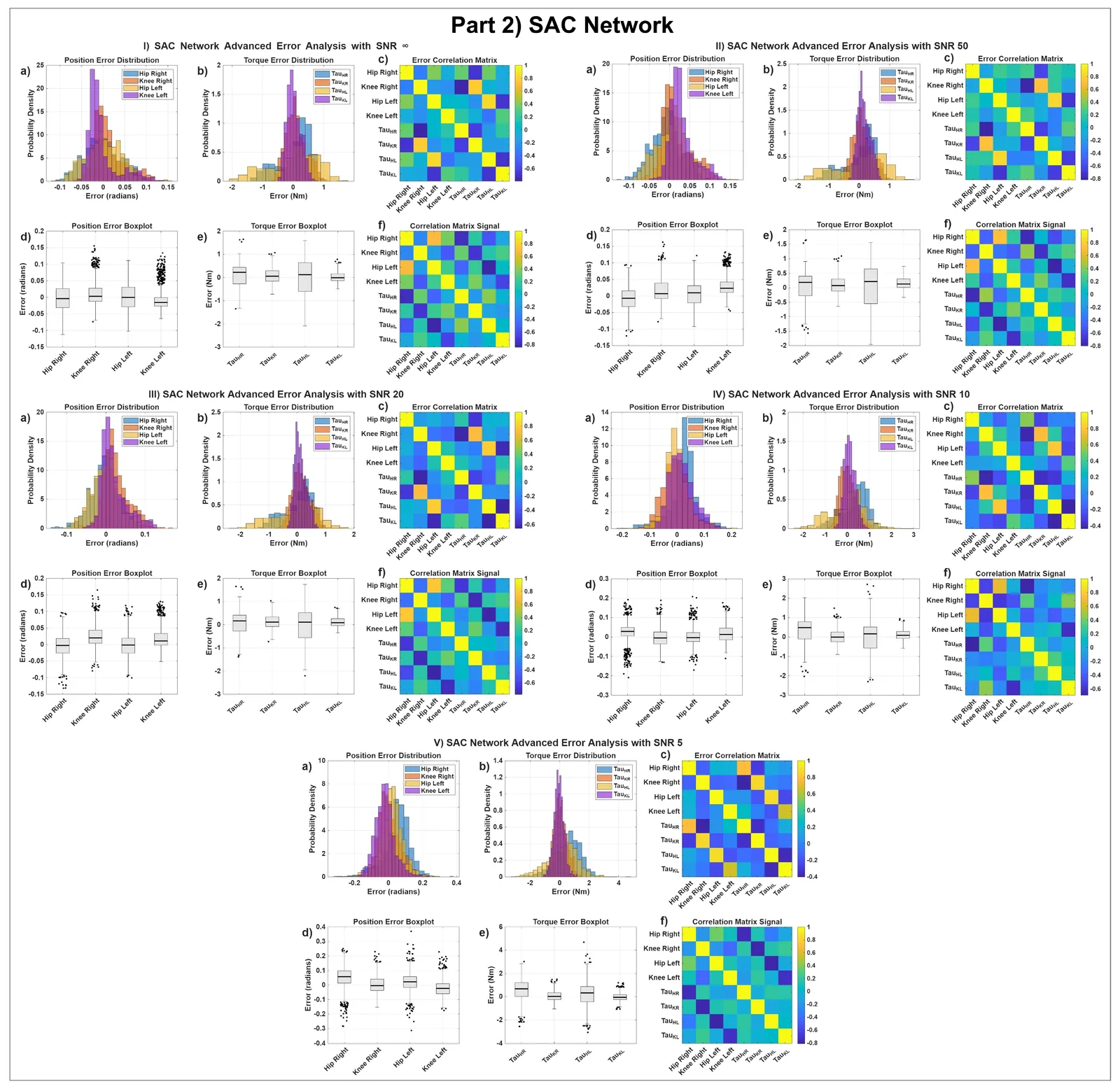

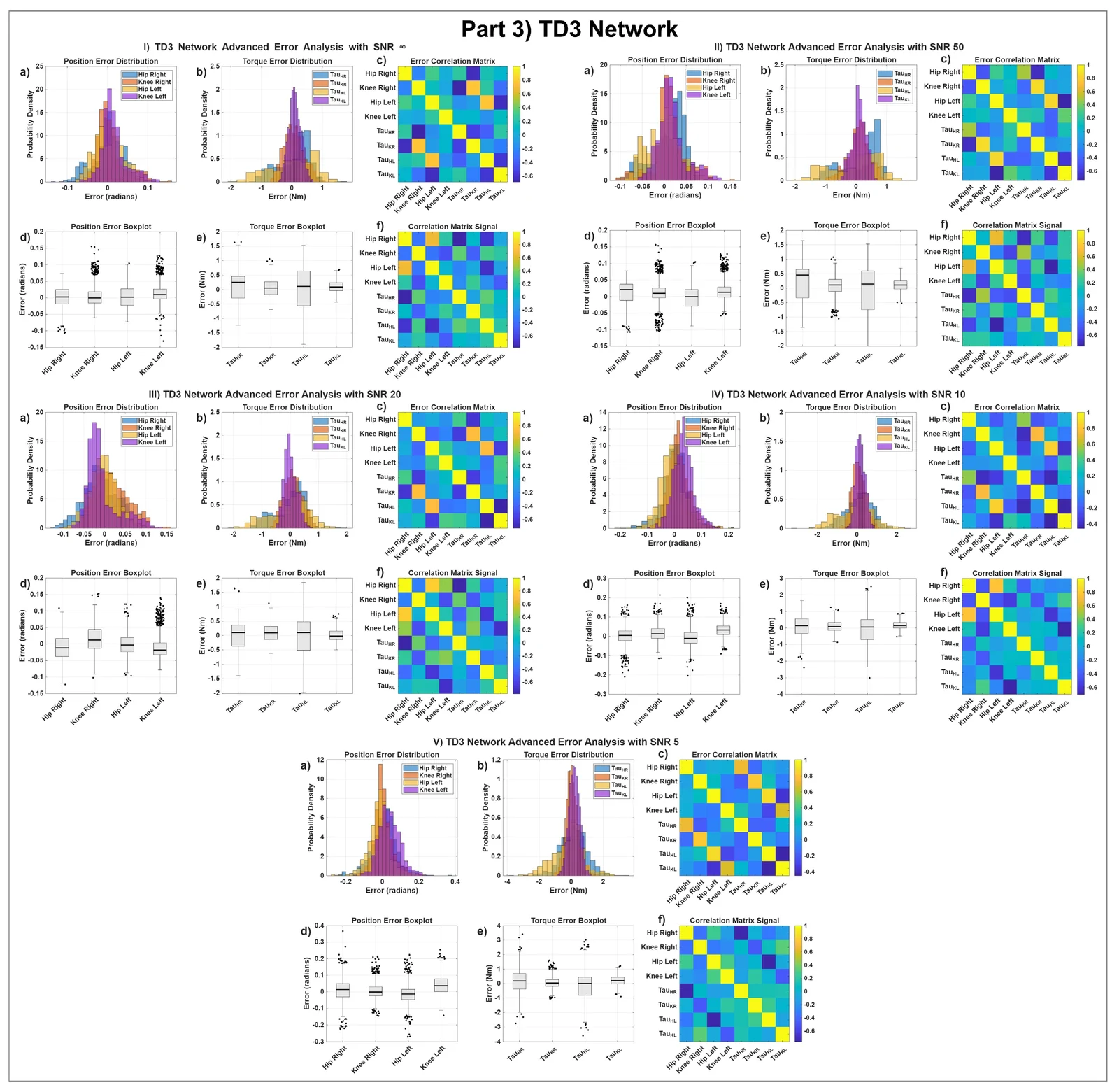
Advanced error analysis of DDPG, TD3, and SAC networks under different SNR conditions. Part (1) corresponds to DDPG, Part (2) to SAC, and Part (3) to TD3. The networks reconstruct joint positions and torques under SNR levels of 5, 10, 20, 50, and ∞ dB (V, IV, III, II, and I, respectively). Panels (**a**,**b**) present the position and torque error distributions, panels (**c**,**f**) show the error and signal correlation matrices, and panels (**d**,**e**) present boxplots comparing the position and torque error distributions for the hip and knee joints of both legs.

To gain deeper insights into the statistical characteristics and robustness of the learned policies, an advanced error analysis was performed on both joint-angle and torque estimations. Figure A1 in Appendix A presents the error distributions, probability density functions, boxplots, and correlation matrices for DDPG, SAC, and TD3 across different SNR levels.

The position error distributions (Figure A1) reveal that TD3 and DDPG exhibit the most concentrated error distributions centred near zero, with relatively low variance across all joints. TD3 particularly demonstrates superior consistency, as evidenced by tighter boxplots and lower outlier presence even at lower SNR values. SAC shows acceptable performance but with broader distributions and more pronounced tails under high noise (SNR = 5 dB). The correlation matrices indicate strong positive correlations between the predicted and actual joint positions for TD3, DDPG, and SAC (values mostly above 0.9 at high SNR), which gradually weaken with increasing noise. This confirms good phase synchronisation of the learned gait patterns.

Torque error analysis (Figure A1) proves more challenging than position tracking. While TD3 and DDPG still maintain relatively centred distributions, the torque errors show higher variance and more outliers compared to angle errors. TD3 continues to demonstrate the best overall behaviour with more symmetric distributions and fewer extreme values. SAC exhibits heavier tails in torque error distributions, particularly at SNR = 5 and 10 dB. The error correlation matrices for torque show moderate correlations (typically 0.3–0.5 for the best algorithms), highlighting the inherent difficulty in precise torque estimation due to the dynamic nature of the human–exoskeleton interaction and sensitivity to model uncertainties.

### 4.5. Ablation Study on Biological Factors

The objective of this ablation study is to quantify the contribution of incorporating patient-specific biological parameters, including bone lengths and muscle masses, into the proposed adaptive control framework. This section is not intended to compare different reinforcement learning algorithms. Based on the results presented in Section 4.2, Section 4.3, Section 4.4 and Section 4.5, TD3 demonstrated the best overall performance in terms of learning stability, tracking accuracy, and robustness to noise. Therefore, TD3 was selected as the representative algorithm for the ablation analysis. Restricting the ablation experiments to TD3 avoids unnecessary computational overhead while providing a controlled evaluation of the effect of biological-parameter integration on controller performance.

Three TD3 variants were evaluated under identical training conditions using 500 training episodes and five independent random seeds at SNR = ∞ dB. The variants were designed to progressively isolate the effects of biological variability and its explicit integration into the learning process, while the results are summarised in Table 4:

- **Baseline:** biological parameters were completely excluded from both the state observation and reward function.
- **Randomisation-only:** biological parameters were randomised during training (domain randomisation) but not explicitly included in the state or reward.
- **Proposed (full integration):** biological parameters were both randomised during training and explicitly incorporated into the agent’s state space and reward function.

The Proposed variant, which integrates biological features directly into the state space and reward function, achieved the best performance across all metrics. It improved the mean reward by approximately 18% compared to the Baseline and 10.5% compared to the Randomisation-only variant. More importantly, it reduced angle tracking error by 27% relative to the Baseline and 15% relative to Randomisation-only. Torque estimation error was also significantly lowered (over 30% improvement compared to Baseline).

Overall, the ablation results indicate that explicit integration of patient-specific biological parameters into the state representation and reward function provides a measurable performance advantage beyond the robustness gained through domain randomisation alone. By conditioning the learning process on individual differences in anthropometry and muscular characteristics, the proposed approach enables the controller to adapt its policy to patient-specific physical properties. This capability is particularly important for personalised lower-limb exoskeleton rehabilitation, where variations in patient morphology and physical characteristics can substantially affect the human–robot interaction dynamics and the effectiveness of assistance.

### 4.6. Sensitivity Analysis to Anthropometric Parameters

**Figure 7:**
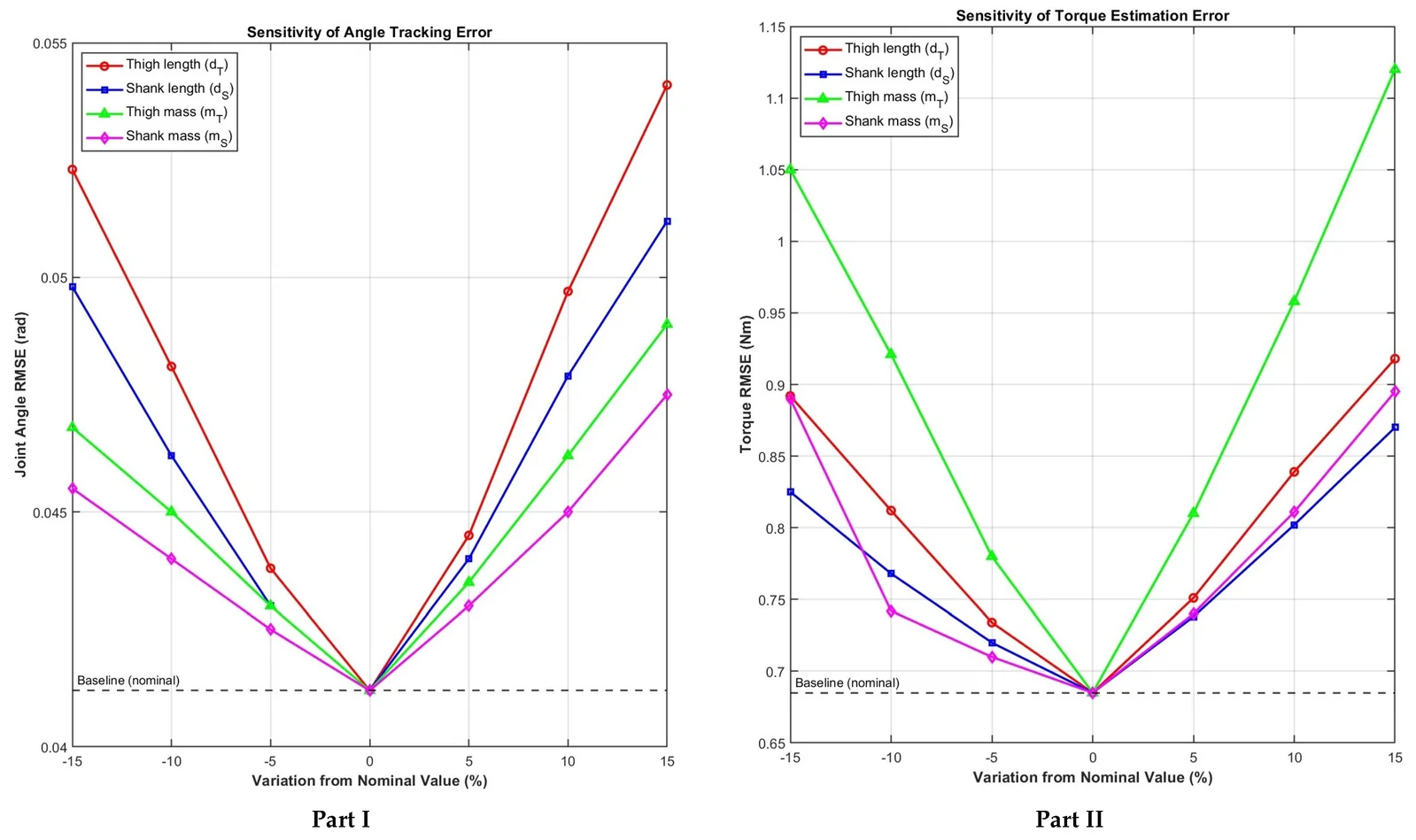
Sensitivity of the TD3 policy to variations in anthropometric parameters. (**Part I**) shows joint angle RMSE (rad), and (**Part II**) shows torque RMSE (Nm). Results represent mean values over 100 test episodes at SNR = ∞ dB. Dashed horizontal lines indicate the nominal-parameter performance. Solid curves represent variations in thigh length ($d_{t}$), shank length ($d_{s}$), thigh mass ($m_{t}$), and shank mass ($m_{s}$).

To assess the robustness of the best-performing TD3 policy to variations in patient-specific anthropometric parameters, a sensitivity analysis was conducted by independently varying thigh length ($d_{t}$), shank length ($d_{s}$), thigh mass ($m_{t}$), and shank mass ($m_{s}$) from −15% to +15% relative to their nominal values reported in Table 1. During each evaluation, the remaining parameters were kept fixed at their nominal values. The policy was evaluated over 100 test episodes at SNR = ∞ dB, and the resulting joint angle RMSE and torque RMSE are presented in Figure 7. The dashed horizontal lines indicate the corresponding performance at the nominal parameter values, with an angle RMSE of approximately 0.0412 rad and a torque RMSE of approximately 0.685 Nm.

Overall, the TD3 policy exhibits a relatively smooth sensitivity profile around the nominal parameter values, although the magnitude of performance degradation varies across the anthropometric parameters. Variations in limb lengths, particularly thigh length ($d_{t}$), have a noticeable effect on both tracking and torque performance. A ±15% variation in thigh mass ($m_{t}$) produces a pronounced increase in torque RMSE, reaching approximately 1.15 Nm compared with the nominal value of 0.685 Nm. Shank length ($d_{s}$) produces a somewhat smaller but still measurable effect on both metrics. This sensitivity is consistent with the influence of limb segment lengths on the geometric and dynamic characteristics of the lower-limb system, including moment–arm relationships.

Variations in segment masses primarily affect torque performance. In particular, a +15% variation in thigh mass ($m_{t}$) increases the torque RMSE by approximately 35–40%, reflecting the influence of segment mass on the inertial and gravitational characteristics of the biomechanical model. In comparison, the effect of mass variation on joint-angle tracking is smaller, with the angle RMSE increasing by approximately 10–14% over the ±15% variation range. The shank mass ($m_{s}$) exhibits a similar but comparatively moderate influence on the control performance.

For small-to-moderate parameter deviations of approximately ±5%, both angle and torque tracking errors remain relatively close to their nominal values. As the deviations increase toward ±10% and ±15%, performance degradation becomes more pronounced, particularly for torque tracking. Nevertheless, the policy maintains stable performance across the entire parameter range evaluated in this study. This indicates that the combination of parameter randomisation during training and explicit incorporation of anthropometric information into the learning framework provides a degree of robustness to variations in patient-specific biomechanical characteristics.

The ±15% variation range was selected to provide a controlled assessment of the policy under moderate deviations from the nominal anthropometric parameters while maintaining stable training and evaluation conditions. However, this range may not represent the full extent of anthropometric and biomechanical variability encountered in clinical populations. In particular, individuals recovering from stroke may exhibit substantial inter-subject differences in muscle mass, limb characteristics, strength, and neuromuscular function due to factors such as muscle atrophy, abnormal muscle tone, prolonged disuse, and unilateral impairment. Therefore, the sensitivity results should be interpreted as an evaluation of robustness within the tested parameter range rather than as a comprehensive characterisation of all clinically relevant patient variability. Future studies will investigate wider parameter ranges and patient-specific scaling strategies based on clinical anthropometric measurements and biomechanical datasets. Such extensions may provide a more comprehensive assessment of policy robustness and further improve the adaptation of the exoskeleton controller to the heterogeneous physical characteristics of rehabilitation patients.

## 5. Discussion

This study investigated a deep reinforcement learning (RL) framework for personalised adaptive control of a lower-limb exoskeleton. The proposed framework combines model-based impedance control with DDPG, TD3, and SAC while incorporating patient-specific biological parameters into the RL state representation and reward function. Unlike supervised sequence-learning methods such as Long Short-Term Memory (LSTM) and Time Delay Neural Networks (TDNNs), which primarily learn temporal dependencies for trajectory prediction, RL formulates exoskeleton control as a sequential decision-making problem. This enables the controller to optimise long-term control performance while considering tracking accuracy, control effort, smoothness, and robustness to disturbances.

Evaluation across SNR levels from 5 dB to ∞ dB demonstrated different characteristics among the three algorithms. TD3 provided the most consistent overall performance in terms of learning stability, tracking accuracy, and robustness, achieving a mean reward of −354.24 at SNR = ∞ dB. DDPG achieved the lowest angle RMSE under noise-free conditions (0.0369 rad), whereas SAC demonstrated comparatively strong robustness under severe noise. Overall, angle RMSE values generally remained below 0.1 rad, with correlation coefficients reaching 0.9825. Torque estimation was more challenging, with RMSE increasing to 1.0591 Nm at SNR = 5 dB, reflecting the greater sensitivity of torque control to dynamic uncertainties and measurement noise.

The ablation study demonstrated the contribution of explicitly incorporating biological parameters into the learning process. The full-integration configuration improved angle RMSE by approximately 27.2% and torque RMSE by 30.5% compared with the baseline without biological information. Domain randomisation alone also improved performance, but the additional gains obtained through explicit integration indicate that providing patient-specific information directly to the learning process can further improve control performance.

The sensitivity analysis showed that the TD3 policy maintained stable performance within the tested ±15% anthropometric variation range. Thigh length and mass had relatively stronger effects on performance, particularly for torque tracking, while small-to-moderate parameter variations resulted in limited degradation. These findings suggest that the proposed framework provides a degree of robustness to moderate patient-specific biomechanical variability.

### 5.1. Limitations

Several limitations should be considered. First, the evaluation was entirely simulation-based using a simplified 2-DoF rigid-body dynamic model for each leg. The model does not fully represent musculoskeletal dynamics, muscle activation, passive joint compliance, soft-tissue deformation, actuator non-linearities, or complex human–exoskeleton interaction forces. Consequently, the sim-to-real gap remains an important challenge for practical deployment. Second, anthropometric randomisation was limited to ±15%, which may not capture the full range of variability and asymmetry observed in some stroke survivors. Third, the reward coefficients were determined through empirical tuning and may not provide an optimal balance across different patients and rehabilitation tasks. Fourth, the current framework relies primarily on biomechanical and anthropometric information and does not incorporate physiological signals or direct movement-intention measurements. Finally, the policies were developed and evaluated using healthy gait reference trajectories, and the ablation analysis was restricted to TD3. Therefore, the present results should be considered a simulation-based proof of concept rather than definitive validation for clinical populations.

### 5.2. Future Work

Future research will focus on experimental validation using a physical lower-limb exoskeleton, initially with healthy participants and subsequently with stroke survivors exhibiting different impairment levels. Pathological gait datasets and broader patient-specific anthropometric distributions will be incorporated to improve clinical relevance and evaluate generalisation to unseen subjects. Further work will investigate automated reward-design and online adaptation methods to reduce dependence on empirical parameter tuning and enable adaptation to changes in patient condition, such as fatigue or altered movement patterns. Physiological sensing modalities, including electromyography (EMG), electroencephalography (EEG), and mechanomyography (MMG), will also be explored to incorporate movement intention and neuromuscular information into the human-in-the-loop control framework. In addition, comparisons with supervised sequence-learning approaches such as LSTM and TDNN will provide a more direct evaluation of policy optimisation versus trajectory prediction for rehabilitation control. More realistic musculoskeletal and human–exoskeleton interaction models, together with safe reinforcement learning and computationally efficient deployment methods, will also be investigated. These developments are expected to support robust real-time operation and facilitate the eventual translation of the proposed framework from simulation to practical rehabilitation applications.

## 6. Conclusions

In conclusion, the proposed deep RL framework provides a promising approach for personalised lower-limb exoskeleton control. Among the evaluated algorithms, TD3 achieved the most favourable overall balance of learning stability, tracking performance, and robustness to noise. The ablation and sensitivity analyses demonstrated that explicit integration of patient-specific biological parameters can improve control performance and provide robustness to moderate anthropometric variations. Although further experimental and clinical validation is required, the results provide a foundation for developing adaptive and personalised intelligent control strategies for lower-limb rehabilitation exoskeletons.

## Figures and Tables

**Table 1:** Anthropometric parameters derived from average adult human data.

Parameter	Description	Value	Parameter	Description	Value
$m_{t}$	Thigh mass for right and left	8.5 kg	$I_{t}$	Moments of inertia thigh	0.15 kg·m^2^
$m_{s}$	Shank mass for right and left	4.3 kg	$I_{s}$	Moments of inertia shank	0.08 kg·m^2^
$L_{t}$	Thigh length	0.42 m	$K_{p_{\mathrm{hip}}}$	Position for hip joint	500 Nm/rad
$L_{s}$	Shank length	0.40 m	$K_{d_{\mathrm{hip}}}$	Velocity for hip joint	50 Nm·s/rad
$d_{t}$	Centre of mass distances thigh	0.25 m	$K_{p_{\mathrm{knee}}}$	Position for knee joint	200 Nm/rad
$d_{s}$	Centre of mass distances shank	0.20 m	$K_{d_{\mathrm{knee}}}$	Velocity for knee joint	100 Nm·s/rad
*g*	Gravity	9.81 m/s^2^			

**Table 2:** Neural-network architecture and training hyperparameters used for DDPG, TD3, and SAC.

Parameter	Value	Parameter	Value
Actor hidden layers	256-128-64	Critic hidden layers	256-128-64
Activation function	ReLU	Output activation	tanh
Optimiser	Adam	Actor learning rate	$10^{-4}$
Critic learning rate	$10^{-3}$	Mini-batch size	128
Replay buffer size	$10^{6}$	Discount factor (γ)	0.99
Soft update factor (τ)	0.01	L2 regularisation	$10^{-4}$
Training episodes	500		

**Table 3:** Summary performance of RL algorithms at selected SNR ∞ dB and 5 dB levels (mean ± std, 5 seeds). Metrics include mean reward (mean ± std), angle RMSE base in rad (mean ± std), torque RMSE in Nm (mean ± std), learning ratio, *p*-value.

Algorithm	SNR (dB)	Mean Reward	Angle RMSE (Rad)	Torque RMSE (Nm)	Learning Ratio	*p*-Value
DDPG	∞	−368.1 ± 22.5	0.0369 ± 0.004	0.712 ± 0.065	0.42	0
TD3	∞	−354.24 ± 18.7	0.0412 ± 0.005	0.685 ± 0.058	0.48	0
SAC	∞	−372.5 ± 25.3	0.0451 ± 0.006	0.734 ± 0.072	0.45	0
DDPG	5	−521.3 ± 41.2	0.112 ± 0.014	1.059 ± 0.098	0.38	0
TD3	5	−498.7 ± 35.6	0.098 ± 0.011	0.978 ± 0.085	0.44	0
SAC	5	−482.4 ± 32.1	0.105 ± 0.012	1.012 ± 0.092	0.51	0

**Table 4:** Ablation study results on the effect of biological parameter integration (mean ± std over 5 seeds, SNR = ∞ dB). Bold values indicate the best performance.

Variant	Mean Reward	Angle RMSE (Rad)	Torque RMSE (Nm)	Notes
Baseline (No variation)	−412.7±28.4	0.0581±0.007	1.284±0.112	Nominal only
Randomisation-only	−378.2±24.1	0.0497±0.006	1.056±0.092	Varied in training, excluded from state/reward
Proposed (Full)	−338.5±19.2	0.0423±0.005	0.892±0.078	Varied + state/reward integration

## Data Availability

The MATLAB implementation of the musculoskeletal model and RL environment is available from the corresponding author upon reasonable request.

## Appendix A. Appendix Results

### Appendix A.1. Detailed Per-SNR Performance

The performance of RL algorithms DDPG, TD3, and SAC at different signal-to-noise ratio (SNR) levels (5, 10, 20, 50, and ∞ dB) is evaluated in Table A1 to assess their effectiveness in controlling lower-limb exoskeletons for stroke rehabilitation. These algorithms provide insights into the robustness, convergence, and adaptability of the algorithms to noise and biological variability. The metrics include mean reward, reward range, final reward, cumulative reward, normalised cumulative reward, learning improvement, learning ratio, and mean rewards across three equal parts of the training process. The analysis reveals distinct performance profiles.

### Appendix A.2. Angle Estimation Performance Metrics

The angle estimation performance of the RL algorithms DDPG, TD3, and SAC was evaluated for controlling a lower-limb exoskeleton across four joints (RH, RK, LH, and LK), as summarised in Table A2. The assessment was conducted under varying SNR levels (5, 10, 20, 50, and ∞ dB) to quantify the robustness of each algorithm to measurement noise and its ability to accurately predict joint angles. The evaluation metrics include Mean Error (ME), Error Standard Deviation (EST), Root Mean Square Error (RMSE), Mean Absolute Error (MAE), *p*-values obtained from normality tests, and the Correlation Coefficient (CC) between the reference and estimated joint angles. All values were obtained from post-training simulations using gait data sampled at 500 Hz.

### Appendix A.3. Torque Estimation Performance Metrics

The torque estimation performance of the RL algorithms (DDPG, TD3, and SAC) was evaluated for controlling a lower-limb exoskeleton across four joints (RH, RK, LH, and LK), as presented in Table A3. The evaluation was performed under different SNR levels (5, 10, 20, 50, and ∞ dB) to assess the robustness of each algorithm in predicting joint torques under noisy conditions. Performance metrics include Mean Error (ME), Error Standard Deviation (EST), Root Mean Square Error (RMSE), Mean Absolute Error (MAE), *p*-values from normality *t*-tests, and the Correlation Coefficient (CC) between the reference and estimated torques. All values were obtained from post-training simulations using gait data sampled at 500 Hz.

### Appendix A.4. Error Distribution Analysis

Figure A1 provides a comprehensive analysis of the deep reinforcement learning algorithms DDPG, TD3, and SAC employed for controlling a lower-limb exoskeleton, focusing on their performance in angle and torque estimation under different signal-to-noise ratio (SNR) conditions (5, 10, 20, 50, and ∞ dB). These visualisations combine the reinforcement learning results with the angle and torque estimation analyses to provide a comprehensive comparison of algorithm performance.

**Table A1:** RL performance metrics across DDPG, TD3, and SAC algorithms at different SNRs. Metrics include mean reward (mean ± std), reward range [min, max], final reward (mean ± std over last 20% episodes), cumulative reward, normalised cumulative reward, learning improvement (second half minus first half mean), learning ratio (second half mean/first half mean), and mean rewards for the first, second, and third thirds of episodes. All values are reported in reward units, with SNR in decibels (dB).

Algorithm	SNR	Mean Reward	Reward Range	Final Reward	Cumulative Reward	Normalised Cumulative Reward	Learning Improvement	Learning Ratio	Mean Reward First Part	Mean Reward Second Part	Mean Reward Third Part
DDPG	5	−929.89 ± 288.80	−3829.50, −720.49	−946.50 ± 341.52	−464,949.26	−929.90	70.22	0.93	−1042.79	−826.52	−921.07
	10	−653.93 ± 264.02	−4166.84, −484.00	−594.30 ± 99.10	−326,966.83	−653.93	148.50	0.80	−815.41	−557.32	−590.04
	20	−487.81 ± 336.70	−4677.66, −303.42	−378.34 ± 35.15	−243,906.22	−487.81	225.30	0.62	−708.78	−375.09	−380.89
	50	−455.18 ± 390.34	−6143.52, −263.11	−360.66 ± 40.51	−227,591.43	−455.18	225.05	0.60	−677.20	−337.11	−352.57
	Inf	−455.71 ± 376.54	−4500.93, −269.85	−338.83 ± 49.83	−227,859.78	−455.72	261.50	0.55	−700.26	−339.20	−329.16
SAC	5	−1298.33 ± 1338.59	−23,142.49, −743.79	−862.93 ± 192.92	−649,165.36	−1298.33	833.39	0.51	−2080.20	−956.61	−862.87
	10	−1088.79 ± 1266.89	−21,234.69, −563.99	−723.02 ± 93.87	−544,399.46	−1088.80	719.38	0.50	−1817.72	−726.43	−726.62
	20	−860.69 ± 1105.08	−12,934.60, −397.72	−477.08 ± 76.58	−430,347.17	−860.69	769.80	0.38	−1614.08	−502.17	−470.35
	50	−770.78 ± 1009.34	−13,355.44, −368.25	−424.55 ± 41.47	−385,391.85	−770.78	695.61	0.38	−1450.79	−443.81	−421.83
	Inf	−837.01 ± 1221.98	−21,027.97, −396.48	−447.52 ± 41.33	−418,509.00	−837.02	747.60	0.38	−1560.52	−499.35	−455.51
TD3	5	−820.43 ± 271.14	−3829.13, −699.63	−789.53 ± 47.86	−410,215.55	−820.43	89.30	0.90	−920.47	−758.37	−783.05
	10	−543.28 ± 150.63	−2213.51, −435.18	−496.44 ± 31.61	−271,640.87	−543.28	95.02	0.84	−641.86	−493.80	−494.78
	20	−389.94 ± 154.34	−2449.62, −283.74	−349.16 ± 20.58	−194,971.67	−389.94	100.93	0.77	−494.24	−332.78	−343.43
	50	−362.43 ± 155.49	−2027.11, −257.27	−331.13 ± 23.76	−181,218.33	−362.44	97.82	0.76	−465.84	−300.39	−321.70
	Inf	−354.24 ± 174.25	−2786.31, −263.14	−303.70 ± 18.14	−177,120.56	−354.24	111.34	0.73	−466.79	−296.45	−300.15

**Table A2:** Angle estimation performance metrics for DDPG, TD3, and SAC algorithms across four joints at different SNRs. Metrics include ME, EST, RMSE, MAE in radians, *p*-value from normality tests, and CC between real and estimated angles. All values are based on 500 Hz gait signal data.

SNR	Metric	DDPG				SAC				TD3			
		RH	RK	LH	LK	RH	RK	LH	LK	RH	RK	LH	LK
5	ME	−0.0125	0.0065	0.0086	0.0457	0.0488	0.0047	0.0208	−0.0215	0.0089	0.0050	−0.0143	0.0404
	EST	0.0745	0.0532	0.0699	0.0564	0.0778	0.0591	0.0686	0.0570	0.0695	0.0502	0.0610	0.0576
	RMSE	0.0755	0.0536	0.0704	0.0726	0.0918	0.0592	0.0717	0.0609	0.0700	0.0504	0.0627	0.0703
	MAE	0.0571	0.0410	0.0541	0.0594	0.0775	0.0469	0.0537	0.0474	0.0534	0.0368	0.0455	0.0549
	*p*-value	0.0000	0.0000	0.0000	0.0000	0.0000	0.0000	0.0000	0.0000	0.0000	0.0000	0.0000	0.0000
	CC	0.9209	0.9310	0.9496	0.9360	0.9415	0.9583	0.9599	0.9365	0.9468	0.9192	0.9557	0.8607
10	ME	0.0251	0.0040	−0.0193	0.0093	0.0234	−0.0031	0.0017	0.0178	0.0014	0.0170	−0.0058	0.0338
	EST	0.0502	0.0449	0.0465	0.0403	0.0511	0.0479	0.0450	0.0459	0.0464	0.0412	0.0491	0.0367
	RMSE	0.0562	0.0451	0.0504	0.0413	0.0561	0.0480	0.0450	0.0493	0.0464	0.0445	0.0495	0.0498
	MAE	0.0452	0.0343	0.0401	0.0312	0.0445	0.0376	0.0326	0.0381	0.0341	0.0333	0.0378	0.0402
	*p*-value	0.0000	0.0000	0.0000	0.0000	0.0000	0.0000	0.0000	0.0000	0.0000	0.0000	0.0000	0.0000
	CC	0.9626	0.9697	0.9653	0.9524	0.9522	0.8626	0.9220	0.9111	0.9286	0.9254	0.9339	0.9610
20	ME	−0.0124	0.027	0.0121	0.0163	−0.0033	0.0265	−0.0016	0.0201	−0.0110	0.0164	−0.0015	−0.0066
	EST	0.0381	0.037	0.0368	0.0311	0.0365	0.034	0.0367	0.0329	0.0405	0.0406	0.0345	0.0392
	RMSE	0.0401	0.0463	0.0387	0.0351	0.0366	0.0431	0.0367	0.0386	0.0419	0.0437	0.0345	0.0397
	MAE	0.0323	0.0339	0.0322	0.0240	0.0284	0.0323	0.0283	0.0261	0.0337	0.0342	0.0269	0.0320
	*p*-value	0.0000	0.0000	0.0000	0.0000	0.0000	0.0000	0.0000	0.0000	0.0000	0.0000	0.0000	0.0000
	CC	0.97203	0.9406	0.9674	0.9574	0.9634	0.9736	0.9733	0.9633	0.9680	0.9167	0.9630	0.9313
50	ME	−0.0018	0.0218	−0.0037	0.0210	−0.0060	0.0177	0.0067	0.0296	0.0087	0.0122	−0.0029	0.0171
	EST	0.0341	0.0336	0.0348	0.0262	0.0384	0.0368	0.0380	0.0296	0.0391	0.0375	0.0372	0.0320
	RMSE	0.0341	0.0401	0.0350	0.0336	0.0388	0.0408	0.0385	0.0418	0.0400	0.0394	0.0373	0.0362
	MAE	0.0263	0.0288	0.0275	0.0240	0.0308	0.0288	0.0314	0.0312	0.0340	0.0275	0.0299	0.0261
	*p*-value	0.0000	0.0000	0.0000	0.0000	0.0000	0.0000	0.0000	0.0000	0.0000	0.0000	0.0000	0.0000
	CC	0.9624	0.9316	0.9740	0.9574	0.9674	0.9650	0.9737	0.9647	0.9585	0.8975	0.9676	0.9099
Inf	ME	−0.0047	−0.0047	0.0143	0.0142	−0.0023	0.0127	0.0003	−0.0048	−0.0003	0.0073	0.0031	0.0156
	EST	0.0366	0.0366	0.0327	0.0340	0.0422	0.0366	0.0407	0.0350	0.0365	0.0345	0.0351	0.0318
	RMSE	0.0369	0.0369	0.0357	0.0369	0.0422	0.0387	0.0407	0.0353	0.0365	0.0353	0.0352	0.0354
	MAE	0.0297	0.0297	0.0242	0.0312	0.0341	0.0272	0.0335	0.0272	0.0284	0.0250	0.0292	0.0244
	*p*-value	0.0000	0.0000	0.0000	0.0000	0.0000	0.0000	0.0000	0.0000	0.0000	0.0000	0.0000	0.0000
	CC	0.9521	0.9485	0.9445	0.9825	0.9645	0.9633	0.9746	0.9396	0.9728	0.9328	0.9646	0.9197

**Table A3:** Torque estimation performance metrics for DDPG, TD3, and SAC algorithms across four joints at different SNRs. Metrics include ME, EST, RMSE, MAE in Nm, *p*-value from normality tests, and CC between real and estimated torques. All values are based on 500 Hz gait signal data.

SNR	Metric	DDPG				SAC				TD3			
		RH	RK	LH	LK	RH	RK	LH	LK	RH	RK	LH	LK
5	ME	−0.0355	0.0644	0.0751	0.2220	0.5751	0.0572	0.1963	−0.0462	0.1770	0.0588	−0.1557	0.2008
	EST	0.7860	0.3874	1.0390	0.3291	0.8898	0.4307	1.0221	0.3397	0.7889	0.3947	0.9739	0.3361
	RMSE	0.7864	0.3925	1.0413	0.3969	1.0591	0.4343	1.0403	0.3427	0.8081	0.3988	0.9858	0.3914
	MAE	0.6014	0.3112	0.8302	0.3219	0.8891	0.3426	0.8289	0.2709	0.6413	0.3048	0.7552	0.3152
	*p*-value	0.0000	0.0000	0.0000	0.0002	0.0000	0.0000	0.0000	0.0000	0.0000	0.0000	0.0000	0.0027
	CC	0.4025	−0.0673	−0.2815	0.2417	0.2499	−0.2176	−0.2733	0.1900	0.3183	−0.1327	−0.3294	0.2095
10	ME	0.3352	0.0536	−0.2064	0.0761	0.3185	0.0259	0.0027	0.1103	0.0987	0.1053	−0.0699	0.1742
	EST	0.6145	0.3667	0.8472	0.2612	0.6136	0.3597	0.8152	0.2557	0.6434	0.3415	0.8657	0.2348
	RMSE	0.6997	0.3704	0.8716	0.2720	0.6911	0.3604	0.8148	0.2784	0.6506	0.3572	0.8681	0.2923
	MAE	0.6097	0.2868	0.6766	0.2111	0.5987	0.2880	0.6457	0.2176	0.5336	0.2805	0.6915	0.2299
	*p*-value	0.0000	0.0000	0.0000	0.0000	0.0000	0.0000	0.0000	0.0229	0.0000	0.0000	0.0000	0.0000
	CC	0.3678	−0.2883	−0.3682	0.1743	0.3845	−0.1082	−0.3102	0.3342	0.2750	−0.1016	−0.3626	0.3109
20	ME	−0.0427	0.1468	0.1059	0.1037	0.0487	0.1424	−0.0312	0.1190	−0.0273	0.1021	−0.0307	0.0125
	EST	0.4802	0.3049	0.7808	0.2233	0.5066	0.3130	0.7618	0.2083	0.5204	0.3217	0.7038	0.2180
	RMSE	0.4819	0.3383	0.7875	0.2461	0.5087	0.3438	0.7621	0.2398	0.5209	0.3374	0.7041	0.2183
	MAE	0.3736	0.2732	0.6701	0.1925	0.4219	0.2710	0.6158	0.1827	0.4199	0.2703	0.5643	0.1752
	*p*-value	0.0083	0.0000	0.0084	0.0000	0.2160	0.0000	0.0001	0.0000	0.1899	0.0000	0.0000	0.0000
	CC	0.5353	−0.0650	−0.4846	0.2395	0.4680	−0.1960	−0.4054	0.3701	0.4620	−0.1251	−0.2493	0.3655
50	ME	0.0631	0.1242	−0.052	0.1222	0.0244	0.1079	0.0515	0.1565	0.1692	0.0856	−0.0446	0.1067
	EST	0.4771	0.3072	0.7649	0.1997	0.5143	0.3128	0.7856	0.2052	0.6266	0.3568	0.7851	0.2308
	RMSE	0.4810	0.3312	0.7664	0.2341	0.5146	0.3308	0.7869	0.2580	0.6487	0.3667	0.7860	0.2542
	MAE	0.4110	0.2654	0.6204	0.1806	0.4224	0.2543	0.6601	0.2007	0.5909	0.2906	0.6623	0.2062
	*p*-value	0.0000	0.0000	0.0000	0.0000	0.0309	0.0000	0.0096	0.0000	0.0000	0.0000	0.0012	0.0000
	CC	0.5233	−0.1609	−0.4903	0.3708	0.4586	−0.1493	−0.4689	0.3552	0.1754	−0.4751	−0.4956	0.1820
Inf	ME	0.0344	0.09446	0.1267	0.1435	0.0591	0.0879	−0.0126	0.0194	0.0781	0.0658	0.0156	0.1019
	EST	0.5434	0.32163	0.7154	0.2009	0.5333	0.3263	0.8089	0.2221	0.5154	0.3271	0.7367	0.1999
	RMSE	0.5443	0.33507	0.7262	0.2468	0.5363	0.3378	0.8086	0.2228	0.5210	0.3335	0.7365	0.2242
	MAE	0.4504	0.26194	0.6363	0.1828	0.4526	0.2637	0.6662	0.1807	0.4535	0.2674	0.6163	0.1786
	*p*-value	0.0070	0.0	0.0014	0.0000	0.0018	0.0000	0.0004	0.0000	0.0000	0.0000	0.0000	0.0000
	CC	0.3731	−0.3063	−0.3322	0.3927	0.4453	−0.2558	−0.4721	0.2904	0.4432	−0.3156	−0.3702	0.4042

## References

[1] Pu L., Wang L., Zhang R., Zhao T., Jiang Y., Han L. (2023). Projected global trends in ischemic stroke incidence, deaths and disability-adjusted life years from 2020 to 2030. Stroke.

[2] Feigin V.L., Brainin M., Norrving B., Martins S.O., Pandian J., Lindsay P., Grupper M.F., Rautalin I. (2025). World Stroke Organization: Global stroke fact sheet 2025. Int. J. Stroke.

[3] Zhang L., Lin F., Sun L., Chen C. (2022). Comparison of efficacy of Lokomat and wearable exoskeleton-assisted gait training in people with spinal cord injury: A systematic review and network meta-analysis. Front. Neurol..

[4] Schofield Z., Gardiner F.W., Bishop L., Spring B., Gale L., Quinlan F. (2023). Best for the Bush In Focus: Heart, Stroke and Vascular Disease.

[5] Foroutannia A., Mohammadian M., Munasinghe K. (2025). A Review of Control Methods in Lower Limb Exoskeleton Robots: From Classical to Machine Learning Approaches. 2025 7th International Congress on Human-Computer Interaction, Optimization and Robotic Applications (ICHORA).

[6] Van der Loos H.M., Reinkensmeyer D.J., Guglielmelli E. (2016). Rehabilitation and health care robotics. Springer Handbook of Robotics.

[7] Bustamante Valles K., Montes S., Madrigal M.D.J., Burciaga A., Martínez M.E., Johnson M.J. (2016). Technology-assisted stroke rehabilitation in Mexico: A pilot randomized trial comparing traditional therapy to circuit training in a Robot/technology-assisted therapy gym. J. NeuroEng. Rehabil..

[8] Ma Z., Wang Y., Zhang T., Liu J. (2025). Reconfigurable exomuscle system employing parameter tuning to assist hip flexion or ankle plantarflexion. IEEE/ASME Trans. Mechatron..

[9] Watanabe Y., Miyazaki T., Wakai Y., Kawashima K. (2026). Gait assist exosuit driven by pneumatic artificial muscles: Integrating posture estimation and assistance via phase-dependent role switching. IEEE Access.

[10] Calabrò R.S., Cacciola A., Bertè F., Manuli A., Leo A., Bramanti A., Naro A., Milardi D., Bramanti P. (2016). Robotic gait rehabilitation and substitution devices in neurological disorders: Where are we now?. Neurol. Sci..

[11] Luo S., Jiang M., Zhang S., Zhu J., Yu S., Dominguez Silva I., Wang T., Rouse E., Zhou B., Yuk H. (2024). Experiment-free exoskeleton assistance via learning in simulation. Nature.

[12] Foroutannia A., Mohammadian M. (2025). A comprehensive survey of lower limb assistive exoskeleton robots: Models, dynamics, mechanics, and control. Rob. Auton. Syst..

[13] Mashud G., Hasan S., Alam N. (2025). Advances in Control Techniques for Rehabilitation Exoskeleton Robots: A Systematic Review. Actuators.

[14] Caulcrick C. (2021). Model Predictive Control for Intelligent Lower Limb Robotic Assistance.

[15] Belal M., Alsheikh N., Aljarah A., Hussain I. (2024). Deep learning approaches for enhanced lower-limb exoskeleton control: A review. IEEE Access.

[16] Coser O., Tamantini C., Soda P., Zollo L. (2024). AI-based methodologies for exoskeleton-assisted rehabilitation of the lower limb: A review. Front. Rob. AI.

[17] Ajayi M.O. (2016). Modelling and Control of Actuated Lower Limb Exoskeletons: A Mathematical Application Using Central Pattern Generators and Nonlinear Feedback Control Techniques.

[18] Foroutannia A., Akbarzadeh-T M.-R., Akbarzadeh A. (2022). A deep learning strategy for EMG-based joint position prediction in hip exoskeleton assistive robots. Biomed. Signal Process. Control.

[19] Nguyen T.T., Nguyen N.D., Nahavandi S. (2020). Deep reinforcement learning for multiagent systems: A review of challenges, solutions, and applications. IEEE Trans. Cybern..

[20] Sivamayil K., Rajasekar E., Aljafari B., Nikolovski S., Vairavasundaram S., Vairavasundaram I. (2023). A systematic study on reinforcement learning based applications. Energies.

[21] Sharifi M., Tripathi S., Chen Y., Zhang Q., Tavakoli M. (2025). Reinforcement Learning Methods for Assistive and Rehabilitation Robotic Systems: A Survey. IEEE Trans. Syst. Man Cybern. Syst..

[22] Duan H. (2024). Reinforcement Learning-Based Control for Bipedal Robots. Ph.D. Thesis.

[23] Liu S. (2024). An evaluation of DDPG, TD3, SAC, and PPO: Deep reinforcement learning algorithms for controlling continuous system. 2023 International Conference on Data Science, Advanced Algorithm and Intelligent Computing (DAI 2023).

[24] Bondre S.V., Thakre B., Yadav U., Bondre V.D. (2024). Deep reinforcement learning algorithms: A comprehensive overview. Deep Reinforcement Learning and Its Industrial Use Cases: AI for Real-World Applications.

[25] Arunkumar S., Jayakumar N. (2025). A comprehensive review on lower limb exoskeleton: From origin to future expectations. Int. J. Interact. Des. Manuf..

[26] Zoss A.B., Kazerooni H., Chu A. (2006). Biomechanical design of the Berkeley lower extremity exoskeleton (BLEEX). IEEE/ASME Trans. Mechatron..

[27] Zoss A., Kazerooni H., Chu A. (2005). On the mechanical design of the Berkeley Lower Extremity Exoskeleton (BLEEX). 2005 IEEE/RSJ International Conference on Intelligent Robots and Systems.

[28] Younis S., Narayan J., Mittal M. (2024). Human-Robot Interaction in Lower Limb Rehabilitation: A Scoping Review. Intelligent Cyber-Physical Systems for Healthcare Solutions: From Theory to Practice.

[29] Parikesit E. (2023). Lower Limb Exoskeleton of Robot Assisted Trainer.

[30] Zhu M., Gong D., Zhao Y., Chen J., Qi J., Song S. (2025). Compliant Force Control for Robots: A Survey. Mathematics.

[31] Cao Y., Ma S., Zhang M., Li Z., Liu J., Huang J., Zhang Z.Q. (2025). Musculoskeletal Model-Based Adaptive Variable Impedance Control With Flexible Prescribed Performance for Rehabilitation Robots. IEEE/ASME Trans. Mechatron..

[32] Zhao Y., Qian K., Bo S., Zhang Z., Li Z., Li G.Q., Dehghani-Sanij A.A., Xie S.Q. (2022). Adaptive cooperative control strategy for a wrist exoskeleton using model-based joint impedance estimation. IEEE/ASME Trans. Mechatron..

[33] Foroutannia A., Ghasemi M. (2023). Predicting cortical oscillations with bidirectional LSTM network: A simulation study. Nonlinear Dyn..

[34] Zhang Z., Wang Z., Lei H., Gu W. (2022). Gait phase recognition of lower limb exoskeleton system based on the integrated network model. Biomed. Signal Process. Control.

[35] Foroutannia A., Akbarzadeh-T M.-R., Akbarzadeh A., Tahamipour-Z S.M. (2023). Adaptive fuzzy impedance control of exoskeleton robots with electromyography-based convolutional neural networks for human intended trajectory estimation. Mechatronics.

[36] Zheng R., Yu Z., Liu H., Lin J., Zeng B., Jia L. (2025). Virtual Impedance Adaptation of Lower-Limb Exoskeleton for Human Performance Augmentation Based on Deep Reinforcement Learning. Chin. J. Mech. Eng..

[37] Sang M.W., Narayan J., Omarali B., Faisal A.A. (2025). Towards Safer Rehabilitation: Improving Gait Trajectory Tracking for Lower Limb Exoskeletons Using Offline Reinforcement Learning. 2025 International Conference On Rehabilitation Robotics (ICORR).

[38] Rose L., Bazzocchi M.C., Nejat G. (2022). A model-free deep reinforcement learning approach for control of exoskeleton gait patterns. Robotica.

[39] Luo S., Androwis G., Adamovich S., Su H., Nunez E., Zhou X. (2021). Reinforcement learning and control of a lower extremity exoskeleton for squat assistance. Front. Rob. AI.

[40] Huang L., Zheng J., Gao Y., Song Q., Liu Y. (2025). A Lower Limb Exoskeleton Adaptive Control Method Based on Model-free Reinforcement Learning and Improved Dynamic Movement Primitives. J. Intell. Robot. Syst..

[41] Luo S., Androwis G., Adamovich S., Nunez E., Su H., Zhou X. (2023). Robust walking control of a lower limb rehabilitation exoskeleton coupled with a musculoskeletal model via deep reinforcement learning. J. NeuroEng. Rehabil..

[42] Zheng R., Yu Z., Liu H., Zhao Z., Chen J., Jia L. (2023). Sensitivity adaptation of lower-limb exoskeleton for human performance augmentation based on deep reinforcement learning. IEEE Access.

[43] Zheng R., Yu Z., Liu H., Chen J., Zhao Z., Jia L. (2023). End-to-end high-level control of lower-limb exoskeleton for human performance augmentation based on deep reinforcement learning. IEEE Access.

[44] Yuan Y., Li Z., Zhao T., Gan D. (2019). DMP-based motion generation for a walking exoskeleton robot using reinforcement learning. IEEE Trans. Ind. Electron..

[45] Yu Z., Zhao J., Chen D., Chen S., Wang X. (2023). Adaptive gait trajectory and event prediction of lower limb exoskeletons for various terrains using reinforcement learning. J. Intell. Robot. Syst..

[46] Dizor R., Raj A., Gonzalez M.B., Smith M.G., Carter Z., Rodrigues M.D., Newton J. (2024). Deep reinforcement learning to assess lower extremity movement intention and assist a rehabilitation exoskeleton. Disruptive Technologies in Information Sciences VIII.

[47] Rv M., Rakshit S. (2024). Deep Reinforcement Learning Based Control of Lower Limb Exoskeleton. 2024 International Joint Conference on Neural Networks (IJCNN).

[48] Xu J., Huang K., Zhang T., Zhao M., Ji A., Li Y. (2024). Mirror adaptive impedance control of multi-mode soft exoskeleton with reinforcement learning. IEEE Trans. Autom. Sci. Eng..

[49] Xu J., Xu L., Ji A., Li Y., Cao K. (2023). A DMP-based motion generation scheme for robotic mirror therapy. IEEE/ASME Trans. Mechatron..

[50] Tu X., Li M., Liu M., Si J., Huang H.H. (2021). A data-driven reinforcement learning solution framework for optimal and adaptive personalization of a hip exoskeleton. 2021 IEEE International Conference on Robotics and Automation (ICRA).

[51] Zhang P., Zhang J., Elsabbagh A. (2023). Fuzzy radial-based impedance controller design for lower limb exoskeleton robot. Robotica.

[52] Aguirre-Ollinger G., Colgate J.E., Peshkin M.A., Goswami A. (2007). Active-impedance control of a lower-limb assistive exoskeleton. 2007 IEEE 10th International Conference on Rehabilitation Robotics.

[53] Li Y. (2017). Deep reinforcement learning: An overview. arXiv.

[54] Arulkumaran K., Deisenroth M.P., Brundage M., Bharath A.A. (2017). Deep reinforcement learning: A brief survey. IEEE Signal Process Mag..

[55] François-Lavet V., Henderson P., Islam R., Bellemare M.G., Pineau J. (2018). An introduction to deep reinforcement learning. Found. Trends Mach. Learn..

[56] Li Z., Guan X., Liu C., Li D., He L., Cao Y., Long Y. (2025). Active Disturbance Rejection Control Based on Twin-Delayed Deep Deterministic Policy Gradient for an Exoskeleton. J. Bionic Eng..

[57] Qasim M.H., Al-Darraji S. (2024). Traversing Dynamic Environments: Advanced Deep Reinforcement Learning for Mobile Robots Path Planning-A Comprehensive Review. Int. J. Comput. Digit. Syst..

[58] Tan H. (2021). Reinforcement learning with deep deterministic policy gradient. 2021 International Conference on Artificial Intelligence, Big Data and Algorithms (CAIBDA).

[59] Dankwa S., Zheng W. (2019). Twin-delayed ddpg: A deep reinforcement learning technique to model a continuous movement of an intelligent robot agent. Proceedings of the 3rd International Conference on Vision, Image and Signal Processing.

[60] Xu Y., Wei Y., Jiang K., Chen L., Wang D., Deng H. (2023). Action decoupled SAC reinforcement learning with discrete-continuous hybrid action spaces. Neurocomputing.

[61] Zhang K., Yang Z., Başar T. (2021). Multi-agent reinforcement learning: A selective overview of theories and algorithms. Handbook of Reinforcement Learning and Control.

[62] Albrecht S.V., Christianos F., Schäfer L. (2024). Multi-Agent Reinforcement Learning: Foundations and Modern Approaches.

[63] Kong S.-H., Nahrendra I.M.A., Paek D.-H. (2021). Enhanced off-policy reinforcement learning with focused experience replay. IEEE Access.

[64] Mao H., Zhang Z., Xiao Z., Gong Z. (2018). Modelling the dynamic joint policy of teammates with attention multi-agent DDPG. arXiv.

[65] Zhang F., Li J., Li Z. (2020). A TD3-based multi-agent deep reinforcement learning method in mixed cooperation-competition environment. Neurocomputing.

[66] Shi H., Wu X., Wang G. (2025). Tracking Control of CSTRs Based on Improved OU Noise and the TD3 Algorithm. IEEE Access.

[67] Lin Q., Ma H. (2023). SACHA: Soft actor-critic with heuristic-based attention for partially observable multi-agent path finding. IEEE Rob. Autom. Lett..

[68] Sumiea E.H., Abdulkadir S.J., Alhussian H.S., Al-Selwi S.M., Alqushaibi A., Ragab M.G., Fati S.M. (2024). Deep deterministic policy gradient algorithm: A systematic review. Heliyon.

[69] Fan Y., Dong H., Zhao X., Denissenko P. (2024). Path-following control of unmanned underwater vehicle based on an improved TD3 deep reinforcement learning. IEEE Trans. Control Syst. Technol..

[70] Ma X., Chen J., Xia L., Yang J., Zhao Q., Zhou Z. (2025). DSAC: Distributional Soft Actor-Critic for Risk-Sensitive Reinforcement Learning. J. Artif. Intell. Res..

[71] Wiltzer H. (2021). On the Evolution of Return Distributions in Continuous-Time Reinforcement Learning.

